# The Relationship between Respiration-Related Membrane Potential Slow Oscillations and Discharge Patterns in Mitral/Tufted Cells: What Are the Rules?

**DOI:** 10.1371/journal.pone.0043964

**Published:** 2012-08-31

**Authors:** Virginie Briffaud, Nicolas Fourcaud-Trocmé, Belkacem Messaoudi, Nathalie Buonviso, Corine Amat

**Affiliations:** Lyon Neuroscience Research Center (CRNL), Team Olfaction: From Coding to Memory, Centre National de la Recherche Scientifique (CNRS) Unité Mixte de Recherche (UMR) 5292 – Institut National de la Santé Et de la Recherche Médicale (INSERM) U1028 - Université Lyon 1, Lyon, France; Instituto de Neurociencias de Alicante UMH-CSIC, Spain

## Abstract

**Background:**

A slow respiration-related rhythm strongly shapes the activity of the olfactory bulb. This rhythm appears as a slow oscillation that is detectable in the membrane potential, the respiration-related spike discharge of the mitral/tufted cells and the bulbar local field potential. Here, we investigated the rules that govern the manifestation of membrane potential slow oscillations (MPSOs) and respiration-related discharge activities under various afferent input conditions and cellular excitability states.

**Methodology and Principal Findings:**

We recorded the intracellular membrane potential signals in the mitral/tufted cells of freely breathing anesthetized rats. We first demonstrated the existence of multiple types of MPSOs, which were influenced by odor stimulation and discharge activity patterns. Complementary studies using changes in the intracellular excitability state and a computational model of the mitral cell demonstrated that slow oscillations in the mitral/tufted cell membrane potential were also modulated by the intracellular excitability state, whereas the respiration-related spike activity primarily reflected the afferent input. Based on our data regarding MPSOs and spike patterns, we found that cells exhibiting an unsynchronized discharge pattern never exhibited an MPSO. In contrast, cells with a respiration-synchronized discharge pattern always exhibited an MPSO. In addition, we demonstrated that the association between spike patterns and MPSO types appeared complex.

**Conclusion:**

We propose that both the intracellular excitability state and input strength underlie specific MPSOs, which, in turn, constrain the types of spike patterns exhibited.

## Introduction

Brain functions involve different cortical rhythms. Among these rhythms, slow oscillations (<10 Hz) and, more specifically, theta oscillations (4–12 Hz), appear to exhibit specific functional roles in the neocortex and hippocampus. Importantly, network theta rhythms demonstrate phase references for discharge activity and/or other cortical oscillations [1], [2]. In such a scenario, these rhythms are thought to underlie a coding strategy for multiple functions, such as sensory processing and memory [3], [4].

The olfactory system is naturally affected by the slow rhythm of respiratory activity, which rhythmically carries odorant molecules to receptor cells. This slow rhythm has been extensively described both at the levels of network and unitary activities in the olfactory bulb, which is the first relay for olfactory information. In network activity, this rhythm appears as a slow oscillation of the local field potential [5]–[7], which patterns the occurrence of fast odor-evoked local field potential oscillations [8]. At the level of the mitral/tufted cells (M/T cells), which are the principal cells of the olfactory bulb, slow respiration-related rhythms are manifested in both spike discharges [9]–[14] and membrane potential fluctuations [15], [16]. Although the slow rhythm’s functional role has never been clearly defined, it is likely a key feature in odor processing. First, previous studies have demonstrated that robust odorant information is transmitted when the spike discharge of a population of M/T cells is integrated over the inhalation phase of the respiratory cycle or over the entire respiratory cycle [17]–[19]. The respiratory cycle thus constitutes a unit of olfactory processing [20]–[22]. Second, membrane potential slow oscillations (MPSOs) have been shown to improve action potential precision [22]. Finally, local field potential respiration-related slow oscillations are thought to facilitate information transfer between the olfactory bulb and olfactory cortex [23], [24].

To date, several patterns of respiration-related discharge synchronizations have been described [13], [24]. Nevertheless, the mechanisms underlying these synchronizations remain unknown. Here, we sought to determine if 1) whether similar rules govern the manifestation of the respiratory patterning of both MPSOs and discharge activity and 2) whether MPSOs and discharge activity are similarly affected by the afferent input and excitability state of the network in which the cell is embedded. To address these questions, we recorded intracellular M/T cell activity in freely breathing anesthetized rats, and we complemented these experimental data with a computational model of the mitral cell. We observed that 1) different types of MPSOs, which can be positive, negative or symmetric, exist and are influenced by sensory afferents, 2) one type of M/T cell spike pattern cannot be associated with any type of MPSO, 3) a single cell can exhibit different MPSO types according to its excitability state, and 4) both the intracellular excitability state and the afferent input strength affect the MPSO type, which in turn constrains the types of exhibited spike patterns.

## Results

**Figure 1:**
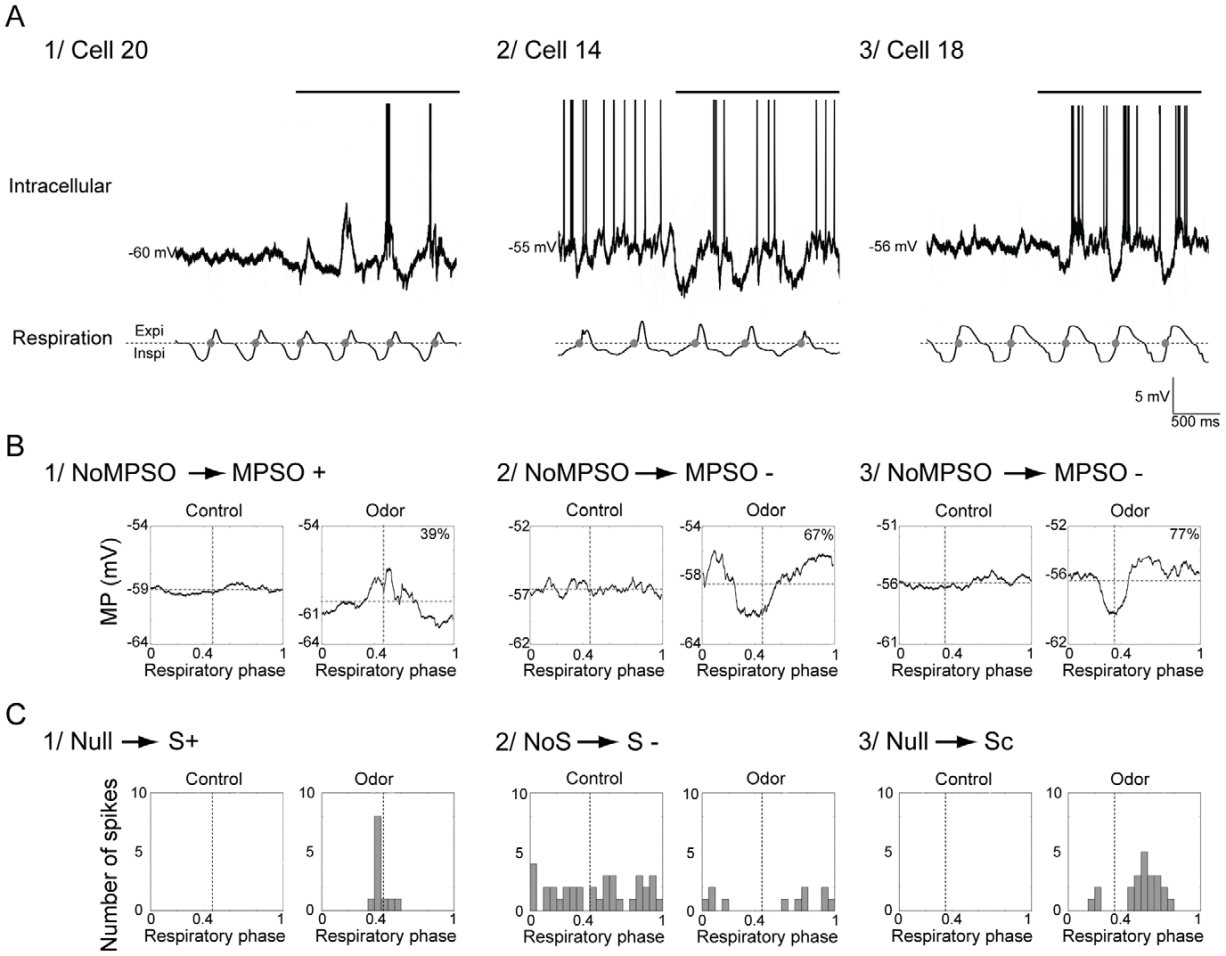
Examples of intracellular activities: membrane potential slow oscillations (MPSOs) and respiration-related discharge patterns. *A) Typical recordings of intracellular signals*. Cell numbers were extracted from the matrix presented in panel B of Figure 2. Top: intracellular recording; bottom: respiratory signal with expiration (Expi) and inspiration (Inspi) separated by a dotted line. The gray circles mark the inspiration/expiration transitions. The horizontal black line at the top of the intracellular signal indicates odor stimulation. The action potentials were truncated to improve the visibility of the membrane potential. The intracellular recordings were obtained without a stabilizing current in 1 and 3 and with a −0.15 nA stabilizing current in 2. *B) Respiration-triggered membrane potential averages before (Control) and during odor stimulation (Odor) of the cells presented in A.* 1) A cell without membrane potential slow oscillation (NoMPSO) prior to odor stimulation acquired a positive membrane potential slow oscillation (MPSO+) during odor stimulation. 2) and 3) Cells without MPSO before odor stimulation that exhibited negative membrane potential slow oscillation (MPSO-) during odor stimulation. A vertical dotted line indicates the transition between inspiration and expiration, and a horizontal dotted line represents the mean membrane potential. The up-point proportion is indicated for each averaged MPSO in the right corner. *C) Respiration-triggered histograms of the action potentials from the cells presented in panel A before (Control) and during odor stimulation (Odor).* 1) A cell without an action potential (null pattern) prior to odor stimulation exhibited an excitatory-simple-synchronized pattern (S+) during odor stimulation. 2) A cell exhibiting a non-synchronized discharge pattern (NoS) in the control condition acquired a suppressive-simple-synchronized discharge pattern (S-) during odor stimulation. 3) A cell with a null pattern during the control period manifested a complex synchronized discharge pattern (Sc) during the odor period. The vertical dotted line indicates the inspiration/expiration transition.

Consistent with the few published *in vivo* studies regarding the mammalian olfactory bulb [15], [25], [26], our intracellular recordings clearly indicated that the membrane potential of some M/T cells developed an oscillation that was synchronized with the respiratory cycle on which the discharge activity was superimposed (**Figure 1A**). A simple visual inspection of the intracellular signals revealed that the membrane potential slow oscillation (MPSO) was not a unique signal but could take on different forms (**Figure 1A, B**). Because various discharge activity patterns have been previously described (excitatory pattern S+, inhibitory pattern S-, complex pattern Sc [**24**]), we expected that a clear rule would govern the association between the MPSO type and discharge pattern. We therefore sought to elucidate the rules governing MPSO expression and respiration-related discharge activity.

**Figure 2:**
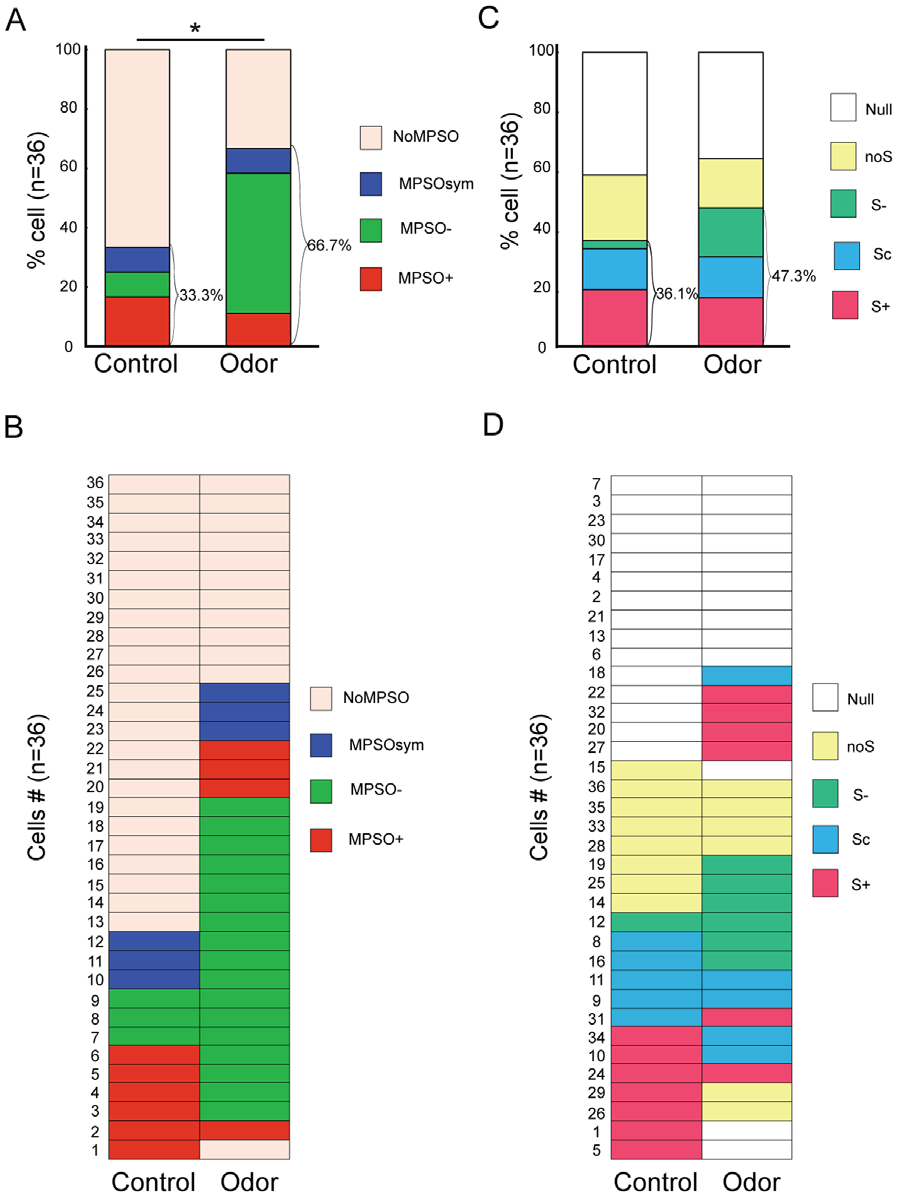
Effects of odor stimulation on membrane potential slow oscillations (MPSOs) and respiration-related discharge patterns. *A) The percentages of MPSO types before (Control) and during odor stimulation (Odor) (n = 36).* The MPSO types are indicated by colors as follows: the positive membrane potential slow oscillation (MPSO+) is in red, the negative membrane potential slow oscillation (MPSO-) is in green, the symmetric membrane potential slow oscillation (MPSOsym) is in blue, and no oscillating signal (NoMPSO) is in beige. According to the χ^2^ statistical test, *p<0.05 between the control and odor period (χ^2^_(1, 72)_ = 6.97). *B) Synthetic view of the MPSO types in the entire population of recorded cells before (Control) and during odor stimulation (Odor) (n = 36).* Each line corresponds to the same cell before and during odor stimulation. The different MPSO types are indicated by the same colors as in panel A. *C) The percentages of respiration-related discharge patterns before (Control) and during odor stimulation (Odor) (n = 36).* The types of respiration-related patterns are indicated by colors as follows: the excitatory-simple-synchronized pattern (S+) is in red, the complex synchronized pattern (Sc) is in blue, the suppressive-simple-synchronized pattern (S-) is in green, the non-synchronized pattern (NoS) is in yellow, and null activity (Null) is in white. *D) Synthetic view of a respiration-related discharge pattern in the entire population of recorded cells before (Control) and during odor stimulation (Odor) (n = 36).* Each line corresponds to the discharge activity pattern of an individual cell before and during the odor stimulation. The depicted cells are the same as those that are presented in panel B. The different types of discharge patterns are indicated by the same colors as in panel C.

Our first aims were to accurately characterize and classify the MPSOs according to precise parameters and to analyze how they were influenced by sensory input. Using autocorrelation and fast Fourier transformation, we observed that 33.3% of M/T cells (n = 12/36) exhibited an MPSO under control conditions (at rest and without odor stimulation; **Figure 2A, B**). The frequency range of these oscillations was 1.91±0.09 Hz (n = 12), which was strongly correlated with the respiratory frequency (Pearson’s correlation coefficient: 0.96). During the control period, the following three MPSO types were present: 1) asymmetric positive MPSOs (MPSO+), which were characterized by a plateau with an upward peak (see the Methods section for details) and were observed in 16.7% of the cells (n = 6/36; **Figure 2B** *Control*); 2) asymmetric negative MPSOs (MPSO-), which were characterized by a plateau and a downward peak and were observed in 8.3% of the cells (n = 3/36; **Figure 2B** *Control*); and 3) symmetric MPSOs (MPSOsym), which were characterized by upward and downward periods that were equivalent in time and were observed in 8.3% of the cells (n = 3/36; **Figure 2B** *Control)*. The remaining 66.7% (n = 24/36) of the cells did not exhibit an observable MPSO during the control period and corresponded to the NoMPSO category.

These proportions were greatly influenced by odorant stimulation. Indeed, we observed that the proportion of cells exhibiting an MPSO significantly increased from 33.3% during the control period to 66.7% during odor stimulation (n = 24/36; p<0.005 using the McNemar test; **Figure 2A**) without a change in frequency (1.99±0.08 Hz, n = 24; Mann-Whitney test: p>0.05). This substantial difference was largely attributed to an increased proportion of the MPSO- type (47.2% during the odor period vs. 8.3% during the control period; χ^2^_(1, 72)_ = 6.975, p<0.05; **Figure 2A, B**). The MPSO+ and MPSOsym types represented 11.1% (n = 4/36) and 8.3% (n = 3/36) of the population, respectively. Interestingly, we did not observe any simple or strict rules for the change in MPSO type during the change from the control to the odor condition. As shown in **Figure 2B**, 45.8% of the cells (n = 11/24) exhibiting NoMPSOs under the control condition continued to demonstrate NoMPSOs when an odor was applied, while the remaining 54.2% (n = 13/24) adopted one of the three MPSO types (**Figure 2B**; examples: **Figure 1A, B**). The cells that exhibited an MPSO- during the control period maintained this MPSO type during odor application. Conversely, the cells exhibiting an MPSO+ during the control period predominantly adopted an MPSO- pattern during odor stimulation (n = 4/6). One of the two other MPSO+ cells maintained its MPSO+ pattern, and the other cell became NoMPSO during odor stimulation. Finally, the three cells presenting an MPSOsym during the control period manifested an MPSO- type during the odor period. This initial analysis revealed that M/T cells can exhibit different MPSO types or NoMPSO, and the influence of the sensory afferent on MPSO expression is complex.

Our second aim was to determine whether these different MPSO types were associated with particular spiking patterns. Specifically, we asked whether MPSO expression strictly conformed with the expression of the respiration-related spiking patterns, which were previously described by Cenier et al. [24]. Indeed, the excitatory S+ pattern is characterized by an increased spike rate at the end of inspiration, and this discharge pattern may be expected to be associated with an MPSO+, with the peaks of the discharge pattern and MPSO+ occurring simultaneously. Similarly, we expected to observe a strict association between the MPSO- and S- patterns, the MPSOsym and Sc patterns, and the NoMPSO and NoS patterns.

To test this possibility, we first needed to characterize the various respiration-related spiking patterns obtained by the intracellular recordings. Examples of respiration-synchronized discharge patterns are presented in **Figure 1A, C**. A typical S+ pattern, in which the action potentials primarily occur within a small time window around the inspiration/expiration transition, was observed in the cell shown in **Figure 1A** **1, C1** (odor period). The S- pattern is displayed in **Figure 1A** **2, C2** (odor period) and is characterized by a decreased discharge activity around the inspiration/expiration transition. The cell shown in **Figure 1A** **3, C3** (odor stimulation) exhibited an Sc pattern, which is defined by multiple peaks/troughs along the respiratory cycle. **Figure 2C** shows that during odor stimulation, the proportion of cells with these respiration-synchronized discharge patterns increased from 36.1% (n = 13/36 during the control period) to 47.3% (n = 17/36). Specifically, we observed that the majority of the cells with respiratory-synchronized discharge patterns during the control period maintained respiratory discharge synchronization during the odor period even if the odor changed the pattern type in some cells (n = 9/13, **Figure 2D**). The proportions of control and odor-evoked respiration-related discharge patterns during the control and odor periods were similar to the results obtained by Chaput et al. [**13**] from a pool of 1408 cells recorded extracellularly, demonstrating that our intracellular sampling was consistent with previous data.

**Figure 3:**
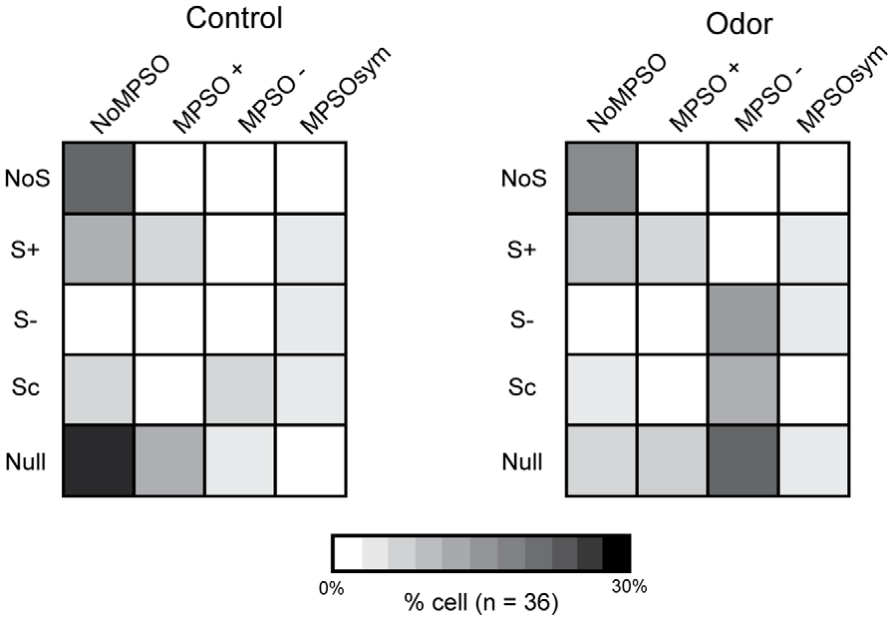
Primary study of the relationship between membrane potential slow oscillations and respiration-related discharge patterns. The percentages of cells exhibiting a specific association between different membrane potential slow oscillation types and various respiration-related discharge pattern types (n = 36) are presented for the control and odor periods. These percentages are indicated in grayscale, in which white represents a null percentage and black indicates 30% of the entire population. The 36 cells that were used to create these matrices are the same cells as those presented in Figure 2. NoMPSO: no oscillation; MPSO+: positive membrane potential slow oscillation; MPSO-: negative membrane potential slow oscillation; MPSOsym: symmetric membrane potential slow oscillation; NoS: non-synchronized pattern, S+: excitatory-simple-synchronized pattern; S-: suppressive-simple-synchronized pattern; Sc: complex synchronized pattern; Null: null activity.

**Figure 4:**
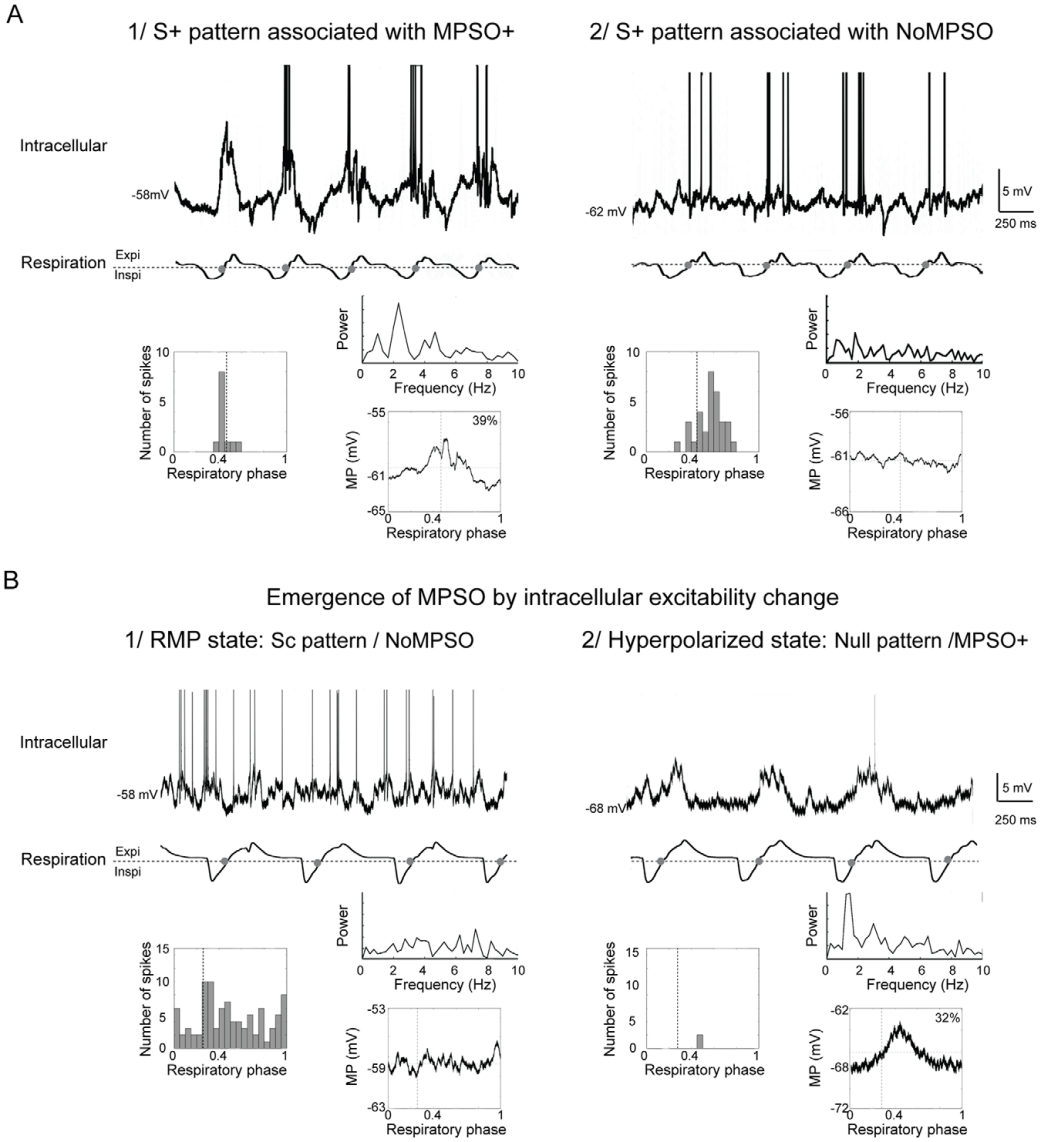
Examples of associations between a respiration-synchronized discharge pattern and different types of membrane potential slow oscillations. *A) Examples of intracellular recordings with a respiration-synchronized discharge pattern that is associated (1) or not associated (2) with a membrane potential slow oscillation (MPSO).* Top: intracellular recording with truncated action potentials; bottom: respiration signal with expiration (Expi) and inspiration (Inspi) separated by a dotted line. The gray circles indicate the signal transitions between inspiration and expiration. Insets: left inset: respiration-triggered histogram of action potentials; right insets: Fourier power spectrum plot of membrane potential (top) and respiration-triggered membrane potential average (bottom). The vertical dotted line indicates the inspiration/expiration transition. The up-point proportion is indicated in the right corner. *B) Example of the emergence of MPSO with an intracellular excitability change.* 1) At the resting membrane potential (RMP; no stabilizing injected current), NoMPSO was observed in association with an Sc pattern. 2) When hyperpolarized (in the H3 state, injected current: −0.2 nA), a positive intracellular oscillation (MPSO+) emerged in association with a null pattern. Top: intracellular recording with truncated action potentials; bottom: respiration signal with expiration (Expi) and inspiration (Inspi) separated by a dotted line. The gray circles indicate the signal transitions between inspiration and expiration. Insets: left inset: respiration-triggered histogram of action potentials; right insets: Fourier power spectrum plot of membrane potential (top) and respiration-triggered membrane potential average (bottom). The vertical dotted line indicates the inspiration/expiration transition. The up-point proportion is indicated in the right corner.

After the spiking patterns were classified, the adequacy between the different MPSO types and different spiking patterns was evaluated. We then constructed matrices crossing the respiration-related discharge pattern type and the MPSO type for each of the 36 cells that were stimulated with an odor (**Figure 3**). Surprisingly, only one strong relationship was observed during both the control and odor stimulation periods; the NoS pattern was always observed in combination with a NoMPSO. The other discharge patterns (S-, Sc, S+ and Null) did not exhibit any exclusive association with a particular MPSO type. In addition, the S- pattern was always associated with an MPSO, which was either an MPSO- or MPSOsym. During the odor period, the S- pattern was predominantly associated with the MPSO- type. The S+ pattern could be associated with an MPSO+ or MPSOsym type, but it was never associated with an MPSO- type. Moreover, cells with an Sc pattern could exhibit an MPSO- or MPSOsym type. The most striking observation was that some respiration-synchronized patterns (S+ and Sc patterns) were associated with NoMPSO. Indeed, the intracellular recording examples illustrated in **Figure 4A** clearly indicate an S+ pattern associated with either an MPSO+, as it would be expected (**Figure 4A** **1**), or with a NoMPSO type (**Figure 4A** **2**).

Furthermore, we were intrigued by the observation that some cells could exhibit a respiration-synchronized discharge pattern while showing no observable MPSO. In this case, how could the discharge be synchronized to respiration? We hypothesized that an MPSO existed in all of the M/T cells that presented a respiration-synchronized discharge pattern but was masked at specific excitability states. This hypothesis would apply when the membrane potential was such that the MPSO amplitude was null or extremely low due to the driving force and conductance levels. Under these conditions, the MPSO would be masked even if the rhythmic input still allowed respiration synchronization of the discharge activity. Modifications to the membrane potential would then reveal such “silent” MPSOs. This possibility was illustrated in **Figure 4B** **,** in which the cell exhibiting an Sc pattern was associated with NoMPSO at the resting membrane potential state and acquired an MPSO at the hyperpolarized state.

The third aim of this study was to explore the aforementioned hypothesis by examining the sensitivity of MPSOs and respiration-related discharge patterns to changes in intracellular excitability.

**Figure 5:**
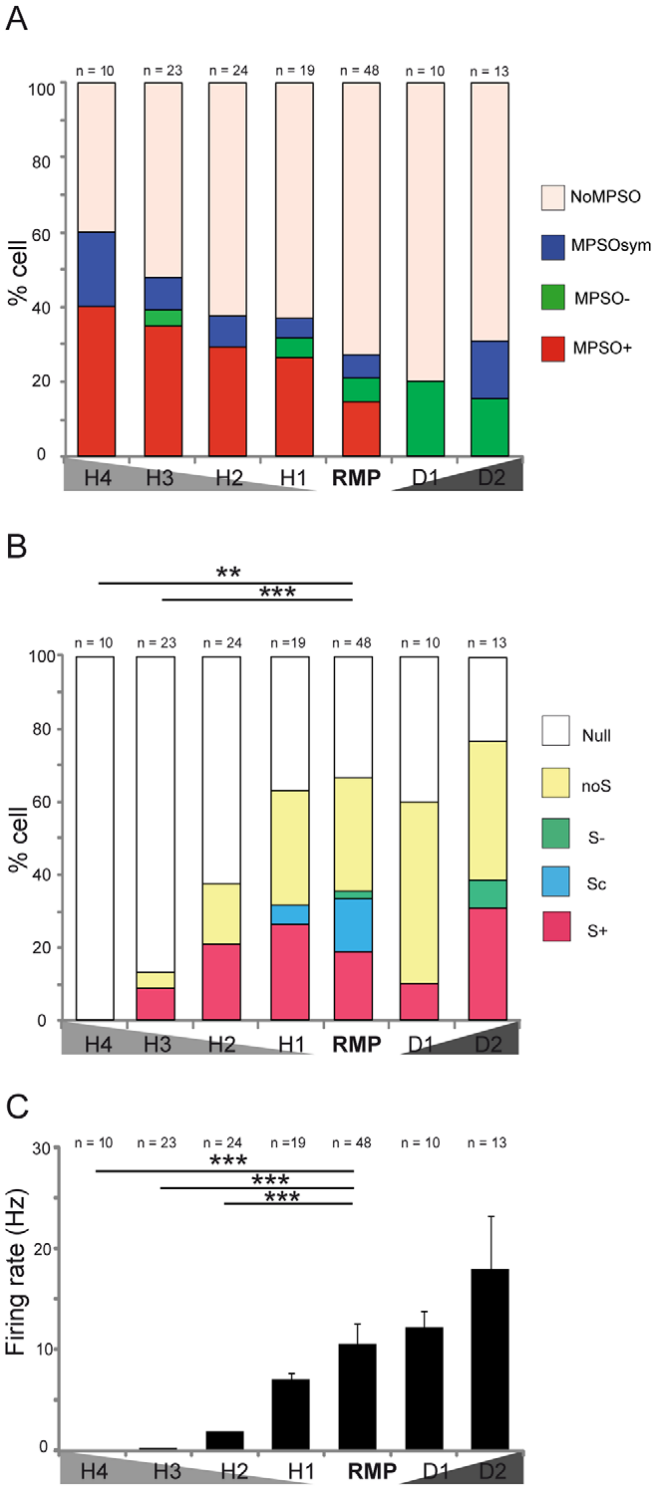
Influence of intracellular excitability state on membrane potential slow oscillations and discharge activities. *A) Percentages of membrane potential slow oscillation types at different excitability levels.* As defined in the Materials and Methods section, H1, H2, H3 and H4 are hyperpolarized states. RMP: resting membrane potential; D1 and D2: the two depolarized states. The number of examined cells at each excitability level is indicated at the top of each bar. The membrane potential slow oscillation types are indicated by color as follows: red indicates a positive membrane potential slow oscillation (MPSO+), green indicates a negative membrane potential slow oscillation (MPSO-), blue indicates a symmetric membrane potential slow oscillation (MPSOsym) and beige indicates the percentage of cells without oscillation (NoMPSO). *B) Percentages of respiration-related discharge pattern types at different excitability levels.* The number of examined cells in each excitability state is indicated at the top of each column. The activity patterns are color-coded as follows: red indicates an excitatory-simple-synchronized pattern (S+), blue indicates a complex synchronized pattern (Sc), green indicates a suppressive-simple-synchronized pattern (S-), yellow indicates a non-synchronized pattern (NoS) and white indicates null activity (Null). According to the χ^2^ statistical test: *p<0.05, **p<0.01 and ***p<0.001. Between RMP and H3: χ^2^_(1, 69)_ = 18,66, p<0.001; between RMP and H4: χ^2^_(1, 56)_ = 14,87; p<0.01. *C) Mean firing rates at different excitability levels.* Each column corresponds to the mean firing rate ± SEM for an intracellular excitability state. The number of cells examined at each excitability level is indicated at the top of each bar. The Mann-Whitney test was used to perform the statistical analysis: *p<0.05, **p<0.01 and ***p<0.001.

First, the probability of the occurrence of the different MPSO types was examined under direct intracellular current injection (as described in the Materials and Methods section). Briefly, we tested 4 hyperpolarization levels (H1, H2, H3 and H4) by injecting progressively negative currents and two depolarized levels (D1 and D2) via positive current injection. The H1 and H2 levels corresponded to a 20% and 50% decrease in discharge activity relative to the spike discharge that was recorded at the resting membrane potential, respectively. The H3 and H4 levels corresponded to potential levels where a few or no action potentials were observed, respectively. The D1 and D2 levels corresponded to a 10% and 30% increase in discharge activity, respectively. The sensitivities of the MPSO types to the intracellular excitability levels are illustrated in **Figure 5A**.

### Hyperpolarized States

Overall, the proportion of MPSO-exhibiting cells increased when the membrane potential was hyperpolarized. In H3, the oscillating cell population represented 47.8% of the cells (n = 11/23), whereas it represented only 27.1% of the cells (n = 13/48) at the resting membrane potential. In addition, the membrane potential hyperpolarization led to an increase in the proportion of cells exhibiting an MPSO+.

### Depolarized States

**Figure 6:**
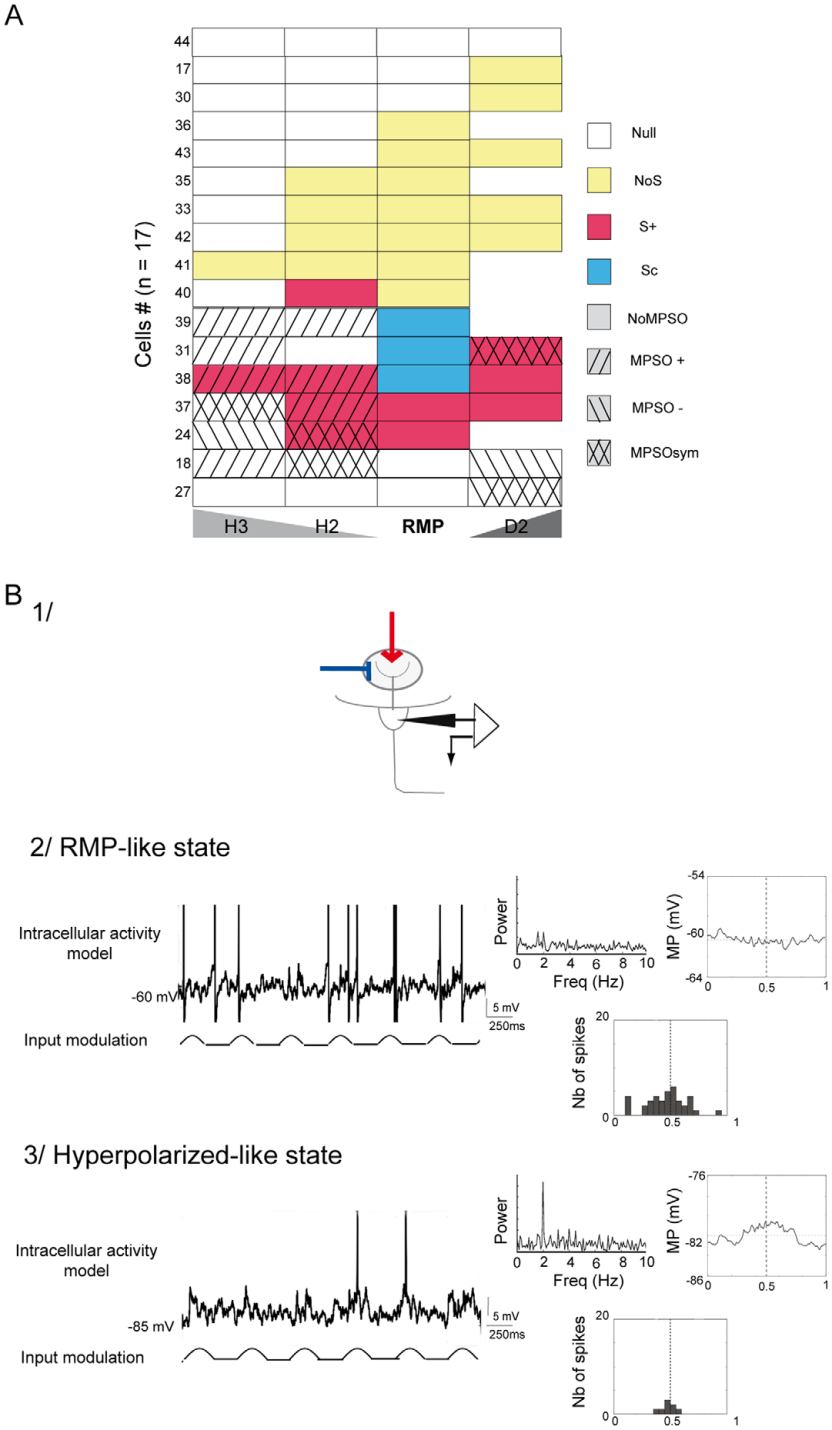
Unmasking of silent membrane potential slow oscillations. *A) A synthetic view of the membrane potential slow oscillations and respiration-related discharge patterns from 17 cells in the H3, H2, RMP and D2 excitability states.* Each line corresponds to a single cell. Of these 17 cells, 9 were also examined with odor stimulation and are indicated with the same numbers (between 1 and 36) as those analyzed in Figure 2. The remaining 8 cells were numbered from 37 to 44. Of the 17 cells, 6 were not depolarized. The activity patterns are indicated by color as follows: blue indicates a complex synchronized pattern (Sc), red indicates an excitatory-simple-synchronized pattern (S+), yellow indicates a non-synchronized pattern (NoS) and white represents null activity (Null). The different types of hatch marks represent the presence and types of membrane potential; the right hatch marks indicate positive membrane potential slow oscillations (MPSO+), the left hatch marks indicate negative membrane potential slow oscillations (MPSO-), the cross hatches indicate symmetric membrane potential slow oscillations (MPSOsym) and a lack of hatches indicates cells without oscillations (NoMPSO). *B) Computational simulation of intracellular activity with rhythmic inputs at the resting membrane potential (RMP-like state, 2) and during negative current injection (hyperpolarized-like state, 3).* 1) Schematic representation of the simulation configuration. The synaptic currents corresponding to a mixture of excitation from olfactory receptor neurons and inhibition from periglomerular cells were injected into the tuft. The simulation parameter details are provided in the Material and Methods section and in **Supporting Information S1**. 2/Simulated recording of a mitral soma model at the resting membrane potential indicating no intracellular oscillations. 3/Simulated recording of a mitral soma model with an additional negative current. This signal showed an MPSO. In 2 and 3: top-left: intracellular activity simulation; bottom-left: simulated input; top-right: Fourier power spectrum plot of the membrane potential and respiration-triggered membrane potential average; bottom-right: the resulting respiration-triggered histogram of action potentials. The vertical dotted line indicates the inspiration/expiration transition.

In contrast to hyperpolarization, the membrane potential depolarization did not appear to affect the frequency of the MPSO occurrence; the proportion of oscillating cells was 30.8% (n = 4/13) in the depolarized state (D2) and 27.1% (n = 13/48) at the resting membrane potential (**Figure 5A**). However, an examination of the MPSO evolution at the single-cell level indicated that the membrane potential depolarization could also trigger an MPSO (**Figure 6A**, hatching). In the depolarized state, the MPSO+ type disappeared in favor of the MPSO- and MPSOsym types (**Figure 5A**).

In addition, we analyzed the effects of intracellular excitability changes on discharge activity. Overall, we observed that the proportions of cells exhibiting S+, S- and Sc were only slightly influenced by excitability changes (**Figure 5B**). We found that the S+ pattern was a particularly stable pattern because it persisted regardless of the excitability state, except in H4 (**Figure 5B** **,** **Figure 6A**). Furthermore, the Sc and S- patterns were rarely present regardless of the excitability level. Indeed, the Sc pattern was observed only in H1 and at the resting membrane potential in a small proportion of cells (H1∶5.3%, n = 1/19; resting membrane potential: 14.6%, n = 7/48; **Figure 5B**). The S- pattern appeared only twice (once at the resting membrane potential and once in D2) (**Figure 5B**). As expected, the more hyperpolarized the cell was, the more frequent the occurrence of the null pattern (**Figure 5B**).

These results suggest that intracellular excitability changes profoundly affected the MPSOs, whereas the respiration-synchronized discharge patterns were only slightly sensitive to the intracellular excitability level. Importantly, this weak effect was not due to a general lack of effect on the generation of action potentials because, as expected, the cells’ firing rates varied with the intracellular excitability state (**Figure 5C**). Indeed, the membrane potential depolarization that was caused by positive current injection led to an increased mean firing rate, whereas progressive hyperpolarization caused by injection with increasing amounts of negative current induced a significant, progressive decrease in the discharge activity (Mann-Whitney test: p<0.001 between the resting membrane potential and the three most hyperpolarized states).

We then combined these new data (i.e., the results obtained from changes in the excitability level) into a matrix in which the MPSO type and spike pattern were represented for each of the 17 cells that exhibited a NoMPSO at the resting membrane potential as recorded under the 4 excitability states (**Figure 6A**). This representation revealed that all of the cells manifesting a non-congruent association (a respiration-synchronized discharge pattern associated with a NoMPSO) at the resting membrane potential developed an MPSO when the intracellular state was modified (**Figure 6A**, cells *24, 31, 37, 38 and 39*). Conversely, all of the cells exhibiting a NoS pattern did not exhibit an MPSO when they were hyperpolarized or depolarized (**Figure 6A**, cells *33, 35, 36, 40, 41, 42 and 43*). Indeed, with the exception of one cell, these cells maintained a NoS/NoMPSO combination or acquired a null pattern associated with a NoMPSO (**Figure 6A**). Taken together, these results indicate that the following are true: 1) the MPSO was actually masked at the resting membrane potential in all of the cases in which we observed an unexpected combination of a synchronized spiking pattern with a NoMPSO and 2) an MPSO is never actually expressed in combination with a NoS pattern.

We then attempted to strengthen our conclusion by using a modeling approach, which allowed us to explore how a silent oscillation may be generated and how it can induce a synchronized discharge pattern (see the Materials and Methods section and **Supporting Information S1** for details). First, we observed that when the cell membrane potential is close to the reversal potential of combined excitatory and inhibitory synaptic inputs, assuming that excitation and inhibition have equal time course, the net synaptic input current is null, which leads to a silent oscillation, characterized by oscillating conductance inputs, and a flat membrane potential. Such a silent oscillation can be unmasked by changing the cell excitability. Second, assuming that larger synaptic conductances are associated with larger conductance noise and thus larger membrane potential noise, we used a one-compartment integrate-and-fire model to demonstrate that the amplitude of membrane potential noise can be oscillatory and entrains a cell rhythmic discharge while the average membrane potential is still relatively flat. We confirmed this result using a modeled mitral cell (see the Materials and Methods section and **Supporting Information S1** for the model’s details) in both a resting-membrane-potential-like state and a hyperpolarized-like state (**Figure 6B**). We examined the resulting activity of the modeled cell (MPSO and spiking activity) when the cell was subjected to rhythmic input in the tuft (**Figure 6B** **1**). As in the simple model, we used a combined synaptic reversal potential that was similar to the cell resting potential. We then observed that, in the resting membrane potential-like state, a 2-Hz rhythmic input resulted in synchronized discharge activity, even in the absence of an MPSO (**Figure 6B** **2**). In the hyperpolarized-like state, the same rhythmic input condition resulted in the appearance of an MPSO and decrease in action potential occurrence (**Figure 6B** **3**). Note that the injection of oscillatory conductances only in the tuft was simpler to fulfill our hypothesis of equal time course for excitation and inhibition, in particular if we take into account the slow time constant observed for lateral dendrite inhibition (50 to 100 ms). This suggests that silent MPSO generation mechanism is mainly located in mitral cell tuft.

Taken together, our experimental and modeling results demonstrated that cells without an observable MPSO but with a respiratory-synchronized discharge pattern likely express a silent oscillation (silent MPSO).

**Figure 7:**
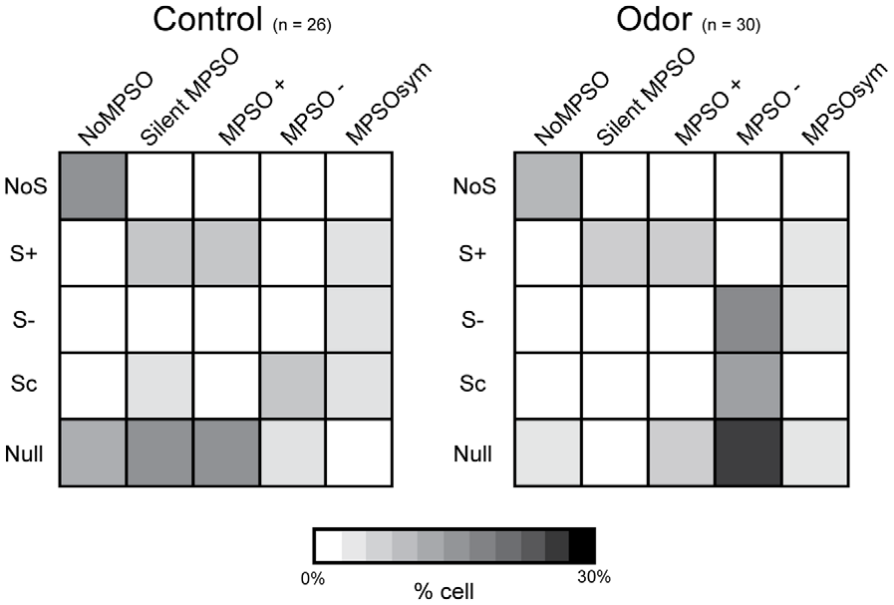
Exclusive associations between membrane potential slow oscillations and respiration-related discharge patterns. The cell percentages exhibiting an association between each type of membrane potential slow oscillation (including silent membrane potential slow oscillations, silent MPSOs) and each type of respiration-related discharge pattern are shown for the control (n = 26) and odor periods (n = 30). The cells that were used to create theses matrices are the same as those used in Figures 2 and 3. For the cells with NoMPSOs or silent MPSOs, only those that were tested using the intracellular excitability protocol were conserved. The cell percentages are represented using a grayscale code, in which white indicates a null percentage and black indicates 30% of the entire population. NoMPSO: no oscillation; silent MPSO: silent membrane potential slow oscillation; MPSO+: positive membrane potential slow oscillation; MPSO-: negative membrane potential slow oscillation; MPSOsym: symmetric membrane potential slow oscillation; NoS: non-synchronized pattern, S+: excitatory-simple-synchronized pattern; S-: suppressive-simple-synchronized pattern; Sc: complex synchronized pattern; Null: null activity.

As a final analysis, we included the silent oscillations revealed by intracellular current injection in matrices similar to those presented in **Figure 3** which already showed that a NoS pattern was always exhibited in combination with a NoMPSO type. **Figure 7** now shows that each type of S pattern is always associated with the occurrence of an MPSO. In addition, **Figure 7** indicates that the S+ pattern was never associated with an MPSO- and the Sc pattern was never associated with an MPSO+. Finally, we observed that olfactory stimulation increased the probability (from 38.5% to 50%) that the M/T cells would exhibit a specific association between a respiration-synchronized discharge pattern and an MPSO.

**Figure 8:**
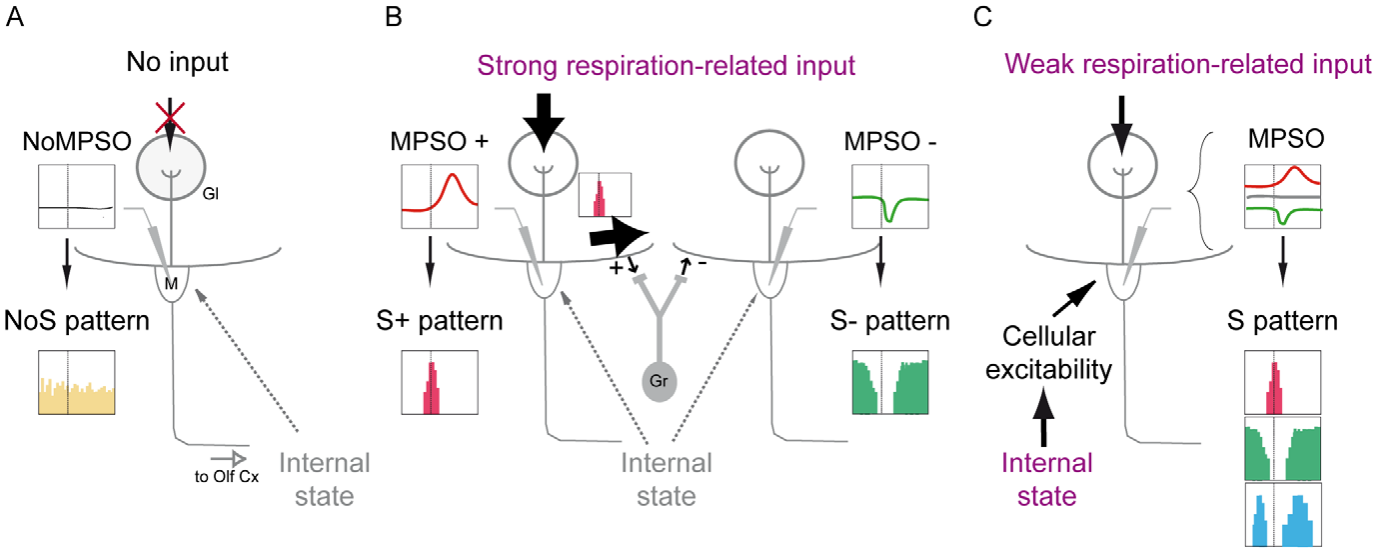
Schematic representation of our data interpretation. *Case A: A mitral/tufted cell related to a glomerular unit receiving no input.* This cell exhibits neither a membrane potential slow oscillation (NoMPSO) nor a respiration-synchronized discharge pattern (NoS). Gl: glomerulus; M: mitral cell; Olf Cx: olfactory cortex. *Case B: A mitral/tufted cell related to a glomerular unit receiving a strong respiration-related input.* This cell exhibits a positive membrane potential slow oscillation (MPSO+) that exhibits an excitatory-simple-synchronized pattern (S+). This S+ spike pattern induces, via granular activation, lateral inhibition of the mitral/tufted cells connected to neighboring glomeruli. Therefore, this cell exhibits a negative membrane potential slow oscillation (MPSO-), which is associated with a suppressive-simple synchronized pattern (S-). Gr: granule cell. *Case C: A mitral/tufted cell related to a glomerular unit receiving a weak respiration-related input.* Because of the weak peripheral input, this cell is strongly influenced by the cellular excitability state, which is dependent on the characteristics of the animal’s internal state (such as neuromodulation and attention). The synergistic effect of the peripheral input and the intracellular excitability can result in several different types of membrane potential slow oscillations (MPSO+, -, sym or silent), which can induce different respiration-synchronized discharge patterns.

In summary, we found that the cells exhibiting an unsynchronized discharge pattern never exhibited an MPSO, whereas the cells with a respiration-synchronized discharge pattern always exhibited an MPSO. Some spiking pattern/MPSO association have never been observed such as S+/MSPO-, S−/MPSO+ and Sc/MPSO+.

## Discussion

The aim of this study was to elucidate the relationship between the respiration-related slow rhythm that was manifested in the M/T cell membrane potential and the M/T cell spike discharge. Specifically, we sought to determine the extent to which afferent sensory inputs and the network’s excitability state influences the expression of both MPSOs and discharge activities. Although some previous studies have reported the existence of slow membrane potential oscillations [15], [16], [22], [25], [26], [27], to our knowledge, our study is the only report characterizing several MPSO types. We observed a complex and intricate relationship between the several MPSO types and respiration-related discharge pattern expressions. However, we conducted an additional analysis that has clarified this issue, and specific relationships were observed between the two activity levels: first, respiration-related synchronized spiking patterns were always associated with an MPSO whereas unsynchronized patterns were never associated with an MPSO; second, as we hypothesized, some spiking pattern/MPSO associations appeared favored (S+/MPSO+, S−/MPSO-) while others were excluded (S+/MPSO-, S−/MPSO+ and Sc/MPSO+). Our data definitively indicate that the respiration-related discharge patterns of the M/T cells are constrained by MPSOs that are, in turn, determined by both sensory inputs and the state of the circuit in which the cell is embedded.

**Figure 9:**
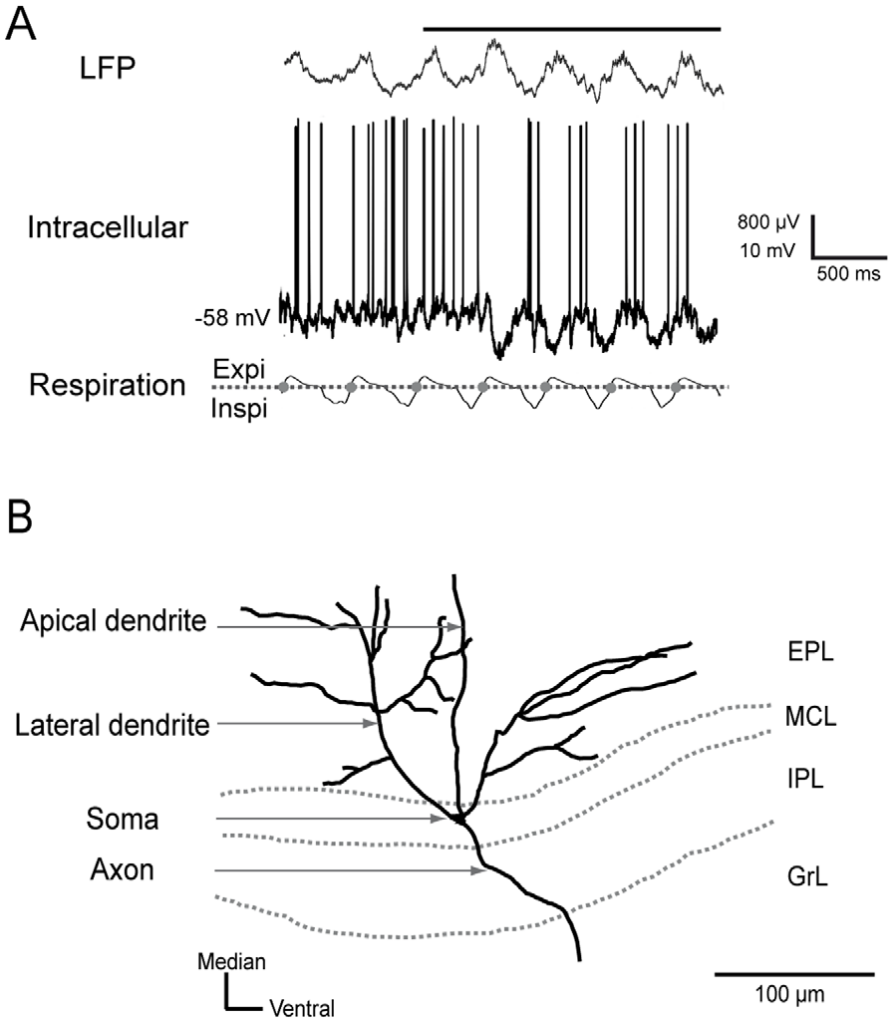
Identification of mitral/tufted cells. *A) Typical example of recorded signals*. The local field potential (LFP) signal in the granular layer was characterized by the presence of a high amplitude wave at the same respiratory frequency at which fast oscillations were superimposed during odor stimulation. The intracellular discharge activity appeared spontaneously and was synchronized to the respiratory rhythm during odor stimulation. Top: LFP signal; middle: intracellular recording; bottom: respiratory signal with expiration (Expi) and inspiration (Inspi) separated by a dotted line. The inspiration/expiration transitions are represented by gray circles. The odor stimulation is indicated by a horizontal black line above the LFP signal. *B) Morphological identification of mitral/tufted cells.* A morphological reconstruction of a mitral/tufted cell recorded *in vivo* and intracellularly labeled with biocytin. Each reconstructed mitral/tufted cell was characterized by the localization of its soma in the mitral cell layer or the deep external plexiform layer, its extensive lateral dendrites and the projection of its apical dendrite into a single glomerulus. EPL: external plexiform layer; MCL: mitral cell layer; IPL: internal plexiform layer; GrL: granular layer.

### The Origin of the Olfactory Bulb Slow Rhythm

The origin of the slow rhythm has been primarily described as peripheral, occurring via the rhythmic stimulation of the olfactory epithelium at each inspiration. Indeed, the local field potential signal has been shown to not exhibit respiration modulation when air flow does not penetrate the nasal cavity [28] or in the absence of mechanical stimulation of the olfactory neuroreceptor [29]. Similarly, the respiratory patterning of the olfactory bulb single-unit activity reflects the phasic stimulation of the olfactory receptors because slow patterning was not observed during continuous odor stimulation [30] or in the absence of air stimulation [28]. Our study demonstrated that the occurrence of respiration-synchronized discharge patterns is enhanced by odor stimulation, which is consistent with the results of previous extracellular studies [13], [31]. Interestingly, we have demonstrated for the first time that these patterns were poorly influenced by intracellular excitability changes, confirming that respiration-related spike activity primarily originates from afferent input activation. However, the origin of MPSOs is not well established. Several studies have proposed an exclusively peripheral origin because MPSOs disappear when no airflow exists in the nasal cavity [22], [25]. However, we observed that odor stimulation modulated the occurrence of MPSOs and improved the synchronization of MPSOs with slow oscillations of the local field potential (data not shown), indicating a common peripheral input.

**Figure 10:**
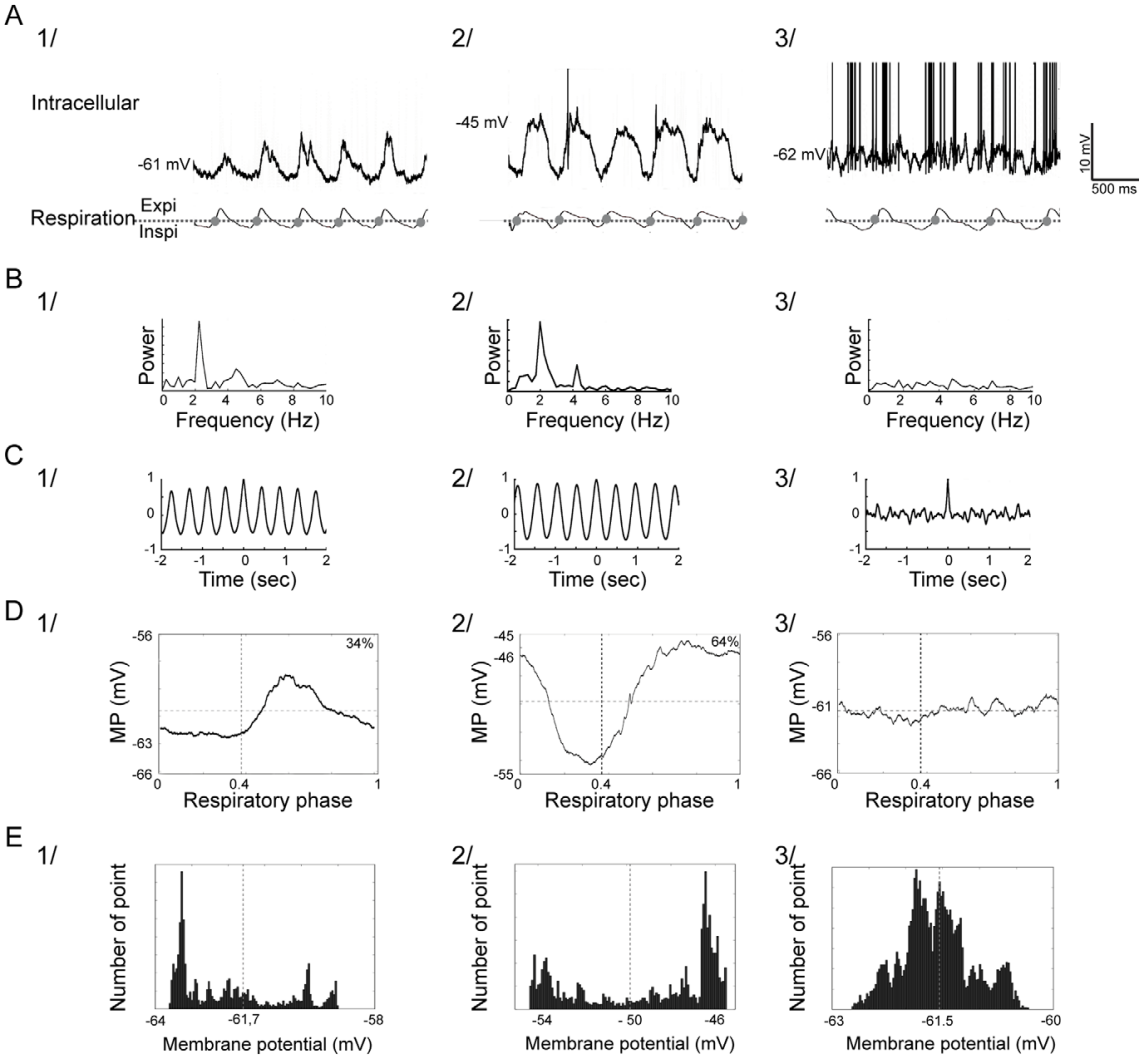
Characterization of membrane potential slow oscillations. A) Intracellular recordings of 1) a cell with a positive oscillation (MPSO+), 2) a cell with a negative oscillation (MPSO -) and 3) a cell without oscillation (NoMPSO). Top: intracellular signal; bottom: respiratory signal with expiration (Expi) and inspiration (Inspi) separated by a dotted line. The gray circles indicate the inspiration/expiration transitions. The action potentials have been truncated to improve membrane potential visibility. B) Membrane potential Fourier power spectrum analysis. Cells with membrane potential slow oscillations (1 and 2) exhibited a higher power in the 2 Hz frequency range (corresponding to the respiratory rhythm) than a cell without oscillation (3). C) Autocorrelograms of the membrane potential. Cells with oscillations (1 and 2) present an oscillating autocorrelogram, while a cell without oscillation (3) shows a flat autocorrelogram. D) Respiration-triggered membrane potential average. A positive membrane potential slow oscillation was characterized by an up-point proportion <42% (1), whereas a negative oscillation manifested an up-point proportion >58% (2) (please refer to the text for details). In the cells without oscillations, the plots were relatively flat. The inspiration/expiration transition is marked by a vertical dotted line, and the mean membrane potential is represented by a horizontal dotted line. E) Histograms of membrane potential distribution. The mean membrane potential is represented by a vertical dotted line.

The peripheral input is not likely the only contributing factor to the slow rhythm; various voltage-dependent conductances also appear to be involved in MPSO expression (See **Figure S1**). Calcium and sodium fluctuations have been shown to reflect slow membrane potential dynamics, and the blockade of some calcium or sodium voltage-dependent conductances result in decreased MPSO amplitude [15], [32]–[34]. In addition, the blockade of some potassium voltage-dependent conductances in olfactory bulb slices has been shown to delay the falling phase of oscillations [33], [35]. Our observation that MPSOs are highly sensitive to excitability changes supports the assumption that MPSOs are also likely shaped by intrinsic components, including voltage-dependent conductances.

### The Relationship between Respiration-related Discharge Patterns and Membrane Potential Slow Oscillations

Our study is the first to dissect the relationships between the various respiration-related discharge patterns of M/T cells and the different types of slow oscillations of their membrane potentials. We demonstrated that the cells that lacked a synchronized discharge pattern never exhibited an MPSO; additionally, S- patterns were always combined with an MPSO- (or MPSOsym), whereas cells with S+ patterns never exhibited an MPSO-. However, some MPSO/discharge activity associations were rather unexpected; for example, the observation of a rhythmic discharge activity, synchronized to the respiratory signal, superimposed onto a non-oscillating membrane potential. We can explain such a discrepancy by our observation that MPSOs can be silenced in some excitability states and revealed in others.

The response patterns of M/T cells are closely correlated with the spatial patterns of glomerular activation. Luo and Katz [27] have demonstrated that M/T cells are robustly excited when their corresponding glomeruli are activated by an odorant but are strongly inhibited when neighboring glomeruli are activated, showing a clear center-surround organization. We used this observation to propose the following interpretation of our data (**Figure 8**). We assumed that the association between MPSO and respiration-related discharge pattern depends on the cell’s location within the spatial pattern of the odor-activated glomeruli. An M/T cell expressing NoMPSO and a spike pattern unrelated to respiration is likely connected to a non-activated glomerulus (either by pure airflow or odor) (**Figure 8A**). In contrast, an M/T cell exhibiting an MPSO is likely related (directly or indirectly) to a rhythmically activated glomerulus (**Figure 8B, C**). A cell located in a strongly activated area receives a strong, direct activating input if the cell is connected to a center-activated glomerulus (**Figure 8B**, left glomerulus) or an indirect inhibiting input if the cell is related to the surrounding glomerulus (**Figure 8B**, right glomerulus). In our model, strong, direct inputs corresponding to excitatory responses were characterized by the presence of an MPSO+. Observations of membrane potential values of MPSO+ induced by odor stimulation in relation to membrane potential values recorded in the control period supported our model. Indeed, the up-value of the MPSO+ is more depolarized than that of the mean membrane potential value of the NoMPSO recorded in the control period, indicating strong excitation (**Figure S1**). This MPSO type has been proposed to be initiated by the activation of AMPA/kainate, NMDA and metabotropic glutamate receptors [15], [22], [25], [32], [36], [37]. The observation that neuroreceptor activation reaches its maximum at the end of inspiration [38] could explain why MPSO+ peaks after the inspiration/expiration transition (data not shown). We additionally demonstrated that MPSO+ imposes an S+ pattern, which constrains the spike discharge to the restricted time window of the inspiration/expiration transition epoch [16], [39]. In this case, the strong afferent input overrides the influence of the cellular excitability, and the spike pattern does not depend on the excitability state. Propagation of the S+ spike discharge along a lateral dendrite may facilitate the rhythmic activation of connected granule cells [16], [25], [40], [41], which could thus induce the rhythmic inhibition of the M/T cells in neighboring glomeruli. This rhythmic inhibition would likely be reflected by an MPSO- (see **Figure S1**) and would impose an S- pattern on these M/T cells, resulting in surrounding inhibition [**27**]. Our model is also supported by the demonstration that the inhibitory discharge responses of M/T cells depend on granule cell-mediated lateral inhibition [42].

In the glomerular maps described by Luo and Katz [27], some glomerular columns are more weakly activated. In this case, M/T cells projecting into such glomeruli are likely more sensitive to intracellular excitability changes (**Figure 8C**). *In vivo*, the cellular excitability state of M/T cells largely depends on an animal’s internal state because neuromodulator systems, such as cholinergic, noradrenergic or serotonergic inputs, act directly or indirectly on M/T cells [43]. According to our results, an M/T cell connected to a weakly activated glomerulus should exhibit a MPSO type which depends on its excitability state. Indeed, we demonstrated that the artificial modification of the intracellular excitability state unmasked the presence of a silent MPSO and led to the modification of the exhibited MPSO type (notably, MPSO+ was promoted by hyperpolarization). We also analyzed the effect of the intracellular excitability state using a mitral cell model. This model confirmed and complemented our experimental results by demonstrating the existence of silent MPSOs, which were predominant at a specific excitability state. Taken together, these data suggest that both the peripheral input strength and the intracellular excitability state determine the MPSO type exhibited by an M/T cell, and the MPSO type constrains the expression of a respiration-related discharge pattern.

### Conclusion

Our M/T cell intracellular recordings obtained from freely breathing anesthetized rats allowed us to demonstrate, for the first time, the specific sensitivity of MPSOs and respiration-related discharge patterns to odor stimulation and intracellular excitability changes. We explained the specific and narrow relationships that exist between these two activity levels. In addition, these specific arrangements can be integrated into the spatio-temporal coding scheme of olfactory processing. We propose that, via dual modulation by input and network excitability, the rough glomerular activation map can be refined as a function of the animal's state.

## Materials and Methods

### Animal Preparation

Male Wistar rats (240 to 350 g, Janvier, Le Genest-Saint-Isle, France) were anesthetized with an intraperitoneal injection of urethane (1.5 g/kg). Local field potential oscillations were used to monitor the depth of anesthesia, and supplemental doses were delivered when necessary. Each animal was immobilized in a stereotaxic apparatus. Respiration was unimpeded and was measured with a homemade bidirectional pressure sensor, which was placed in front of the right nostril, as described by Roux et al. [44]. This sensor measured respiratory airflow changes as a depression during inspiration and a super-pressure during expiration. Viscous xylocaine (2%) was applied to the skin before surgery, and a craniotomy was performed above the dorsal surface of the left olfactory bulb. The dura was removed, and the olfactory bulb was protected by a 3% agar Ringer’s lactate solution. The cisterna magna was then drained. The animal’s temperature was maintained at 37°C with a heating blanket (Harvard Apparatus, Holliston, MA, USA). At the end of the experiments, the rats were euthanized by a lethal intraperitoneal injection of pentobarbital.

All of the experiments were conducted in strict accordance with the European Community Council directive of November 24, 1986 (86/609/EEC) and those of the French Ethical Committee and French Legislation. These experiments were approved by the Ethics Committee of the Université de Lyon 1 (authorization number: BH2008–08).

### *In vivo* Electrophysiological Recordings

Intracellular recordings were performed in the lateral or medial area of the left olfactory bulb using borosilicate glass micropipettes (o.d. = 1.5 mm; i.d. = 0.86 mm; Harvard Apparatus, Holliston, MA, USA) pulled with a horizontal puller (model P-97, Sutter Instruments, Novato, CA, USA). The micropipettes were filled with a solution of 2 M potassium acetate. To allow the morphological identification, we also included 2% biocytin (Sigma, Saint Louis, MO, USA) in the micropipette solution during some of the recordings. The electrode resistances ranged from 55 to 180 MΩ. The electrophysiological signal was amplified and low-pass filtered on-line at 10 KHz by an intracellular amplifier (Axoclamp 2B, Axon Instruments, Foster City, CA, USA). The signal was then digitized at 20 KHz (PCI-DAS 1602/16, Measurement Computing, Norton, CA, USA) and stored on a personal computer using the ELPHY software program (Sadoc G., Centre National de la Recherche Scientifique). During the experiment, the resting membrane potential was recorded with no current injection in 33/48 cells and with small stabilizing currents (−0.14±0.03 nA, mean ± SEM) in 15/48 cells. Intracellular excitability changes were produced by progressive direct current (DC) injections with an intracellular amplifier. The membrane potential was hyperpolarized at 4 distinct levels via negative current injections. The first level, H1, was obtained via a −0.14±0.03 nA (n = 19) DC injection and caused a 20% decrease in discharge activity relative to the spike discharge activity that was recorded at the resting membrane potential. The second level (H2) corresponded to the potential level at which the discharge activity decreased by 50% via a −0.22±0.04 nA DC injection (n = 24). The third and fourth hyperpolarized levels (H3 and H4) were defined as the potential levels that resulted in a level of discharge activity in which few or no action potentials were observed, respectively. These potential levels were obtained using current injections (for H3: −0.30±0.04 nA, n = 23; for H4: −0.41±0.07 nA, n = 10) carrying membrane potentials just below (H3) or well below (H4) the action potential threshold. Two depolarized states were also used to analyze the intracellular excitability effects. D1 corresponded to the level at which a 10% increase in discharge activity was induced and was evoked by a positive DC injection of 0.11±0.02 nA (n = 10). D2 corresponded to a 0.18±0.03 nA injection (n = 13) and induced a 30% increase in discharge activity. The input resistance of the cells was measured by applying brief hyperpolarizing current pulses with a programmable stimulator (Master-8, A.M.P. Instruments LTD, Jerusalem, Israel). Before the impalements were performed, the electrode tip potential was controlled by measuring the DC offset of the electrode in the extracellular medium. This measurement was performed again at the end of the experiment. For all of the cells analyzed, the electrode tip potential was not modified between these two measurements.

The local field potential recordings were performed simultaneously using silicon probes (Neuronexus Technology, Ann Arbor, MI, USA) placed in medial or lateral positions in the left olfactory bulb at a depth of approximately 1500 µm. The broadband signal (0.1 Hz to 5 KHz) was amplified with a homemade amplifier; it was digitized at 20 KHz (PCI-DAS 1602/16, Measurement Computing, Norton, USA) and stored on a personal computer.

### Odor Stimulation and Acquisition Protocol

Odor was delivered in front of the left nostril using a homemade olfactometer linked to a respiratory sensor; odor onset was triggered at the end of an expiration period. Three different odorant molecules, isoamyl acetate, heptanone and ethyl pentanoate, were used separately (Sigma-Aldrich, Saint Louis, MO, USA). A single odor was delivered at 9% of the saturated vapor pressure, and 2 consecutive stimulations were separated by at least 1.5 min. The recording session consisted of 4 seconds of pre-stimulus activity, 3 seconds of odor-evoked activity and 5 seconds of post-stimulus activity (n = 97 recordings with odor stimulation). Fifty 12-second recordings were performed without odor stimulation.

### Histology

Biocytin was injected into some of the recorded cells using 0.2 nA depolarizing current pulses of 500 ms every 1 second for 10 minutes. At least 1 hour after the biocytin injection, the rat was transcardially perfused with Ringer’s solution, followed by a 4% paraformaldehyde solution. After the animal was decapitated, the olfactory bulb was removed and placed in cold 30% sucrose in 0.1 M phosphate buffer. After the freezing process was complete, 80-µm-thick coronal sections were cut with a cryostat (Reichert-Jung, NuBlock, Germany). Biocytin was detected after treatment via the avidin-biotin-peroxidase complex (ABC Elite Kit, Vector Laboratories, Burlingame, CA, USA) and incubation with 3, 3′ diaminobenzidine-tetra-hydrochloride (Sigma-Aldrich, Saint Louis, MO, USA). The sections were stained with cresyl violet, dehydrated and mounted with DPX. The cells were visualized using an optical microscope.

### Data Analysis

Data processing was performed using the OpenElectrophy open-access homemade software program ([45]; freely available at http://neuralensemble.org/trac/OpenElectrophy). This program is associated with a MySQL database, and specific analyses were performed with scripts written in Python.

#### Respiration

We examined respiration-related phenomena, such as respiration-related discharge patterns and membrane potential slow oscillations (MPSOs). Thus, we chose to represent the dataset as a function of the respiratory phase [44]. Briefly, the recorded respiratory signal was processed to extract each respiratory cycle. The time component of each period was then converted into a circular phase component that was defined between 0 and 1, where 0 and 1 represented the beginning of inspiration and the end of expiration, respectively. Using this new reference, the electrophysiological signals were no longer represented as a function of time; they were rather represented as a function of the related respiratory phase. During the phase transformation, inspiration and expiration were scaled differently to place the transition point between inspiration and expiration at the same phase for all of the respiratory cycles. The main advantage of this method was that the phase representation was common to all of the trials, unlike the time representation. The electrophysiological recordings were analyzed relative to the inspiration/expiration transition, which could be automatically detected as the point at which the respiratory signal crossed zero, which corresponded to the point of null airflow in the rising phase.

The respiratory frequency (in Hz) of each recording was calculated by dividing the number of extracted respiratory cycles by the time between the beginning of the first cycle and the end of the last cycle of the total recording time.

#### Intracellular signal

For this analysis, we selected a total of 48 M/T cells with stable membrane potentials and resting membrane potentials of less than −50 mV. Of these 48 M/T cells, 36 were stimulated with odor, and 29 were subjected to at least one excitability state, predominantly the hyperpolarized state, in addition to the resting state. Because depolarized states are more difficult to stabilize, only 13 M/T cells were subjected to these conditions. Cell-type identification was performed according to electrophysiological and anatomical criteria. The recorded M/T cells, for which a typical signal can be observed in **Figure 9A**, exhibited a mean resting membrane potential of −58.09±0.85 mV (n = 48) and an input resistance of 49.43±5.52 MΩ (n = 33). These electrophysiological characteristics have been described as being specific to M/T cells [46]. The electrophysiological identification of the M/T cells was confirmed by anatomical criteria using biocytin staining of 6 M/T cells (**Figure 9B**). These cells were characterized by observations of soma localized to the mitral cell layer or the deep external plexiform layer, an apical dendrite directed toward the glomerular layer, lateral dendrites spreading into the external plexiform layer and an axon in the internal plexiform layer.

#### Discharge activity analyses

We collected the action potential times for all of the intracellular recordings using the built-in spike detection option in the OpenElectrophy software.

#### Respiration-related discharge pattern

As previously described [13], M/T cell activity is characterized by its temporal pattern along the respiratory cycle. To determine this pattern, we constructed respiratory cycle-triggered histograms of the action potentials (20 bins, based only on simultaneously recorded respiratory signals) (see **Figure 1C** and **Figure 4** for examples). Because a respiratory cycle lasts for approximately 500 ms, each bin represented approximately 25 ms. The respiratory cycle-triggered histograms of the action potentials were calculated using 17.34±0.30 (n = 50) respiratory cycles from recordings without odor stimulation, 6.78±0.15 (n = 97) respiratory cycles from the control period of the odor-stimulated recordings and 4.58±0.18 (n = 36) respiratory cycles from the odor period of the recordings. The histograms were classified into the following five different types according to the classifications of Chaput et al. [13]: 1) non-synchronized patterns (NoS) characterized by a uniform distribution of action potentials throughout the respiratory cycle, 2) excitatory-simple-synchronized patterns (S+) presenting a single increase in firing activity synchronized with the respiratory cycle, 3) suppressive-simple-synchronized patterns (S-) presenting a single decrease in firing activity synchronized with the respiratory cycle, 4) complex synchronized patterns (Sc) exhibiting multiple firing increases and/or decreases along the respiratory cycle and 5) null recordings presenting few or no action potentials. The patterns were classified by visual inspection; they were first observed by individuals and were subsequently observed by a group of three observers. The patterns were then selected for further analysis only if they were classified as the same type by at least two of the three observers.

#### Membrane potential slow oscillation analyses

To specifically study the evolution of the membrane potential in M/T cells, we removed action potentials from the membrane potential traces using the following procedure. For each recording, the interspike intervals were calculated, and an interspike interval histogram was used to differentiate single action potentials from a burst of action potentials in our database. In the second step, the averaged single action potentials and the action potential bursts were defined using spike-triggered averaging of the membrane potential. Next, two parameters were measured; first, the time between the first action potential peak and the pre-depolarization onset, *Δt_pre_*, was measured, and second, the time between the last action potential peak and the after-hyperpolarization end, *Δt_post_*, was measured. These times were used to replace the single action potentials or action potential bursts (at time *t_AP_*) with a linear interpolation of the subthreshold membrane potential from *t_AP_ - Δt_pre_* to *t_AP_ + Δt_post_*. This raw intracellular signal, from which the action potentials had been removed, was called the “cut-signal”. Moreover, the raw intracellular signal corresponds to the “cut-signal” for the recordings that lacked action potentials.

From this “cut signal”, the presence of an MPSO was determined using two methods, a fast Fourier transform analysis and an autocorrelation analysis of the membrane voltage. The autocorrelation was calculated using the following equation:
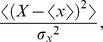
where x represents the signal, <.> represents the mean and σ represents the standard deviation.

To be classified as oscillating, a “cut signal” was required to fulfill two criteria. First, its Fourier spectrum was required to exhibit a clear power peak at the respiratory frequency (between 1 and 3 Hz) and to be three times the standard deviation above the Fourier power spectrum median (computed with a fast Fourier transform over the 0–10 Hz range) (**Figure 10A** **1, 2** and **Figure** **10B** **1, 2**). Second, the autocorrelogram of the cut signal was required to demonstrate a persistent oscillation (not damped during the entire autocorrelation window) (**Figure 10C** **1, 2**). If both criteria were not fulfilled, the trial was classified as non-oscillating (**Figure 10A** **3, B3, C3**). When the two criteria resulted in an opposite conclusion, the trial was not retained for further analysis. The Fourier power spectrum peak defined the MPSO oscillatory frequency value, which was confirmed by the autocorrelogram.

The average intracellular oscillations were not symmetric and regular like typical sine waves. We therefore created specific analyses to classify these oscillations depending on the shape of their average cycles. The “cut signal” was averaged across several respiratory cycles (the same number of respiratory cycles that was used for the respiratory cycle-triggered spike histograms) to obtain the respiration-triggered membrane potential average (**Figure 10D**). From this averaged signal, we extracted the median value from the most negative 30% of the membrane potential points (NEG-med) and the median value from the most positive 30% of the membrane potential points (POS-med). We then defined the mean value of the membrane potential oscillation as OSCmean =  (NEG-med + POS-med)/2. The membrane potential values below OSCmean were defined as down-points, and those above were defined as up-points. The median, mean and point proportions were also visualized with histograms of the membrane potential distribution (**Figure 10E**). When the up-point proportion was <42%, which indicated a plateau and an upward peak, the signal was classified as positive (MPSO+, **Figure 10D** **1, E1**). In contrast, if the up-point proportion was >58%, which indicated a downward peak, the MPSO was classified as negative (MPSO-, **Figure 10D** **2, E2**). When the up-point proportion was between 42% and 58%, we performed the same analysis cycle by cycle. Indeed, in some cases, one of the cycles was clearly an outlier, or there could be a slow drift in the potential, resulting in a misleading global average. For these cases, if the cycle-by-cycle classification was coherent over more than 80% of the cycles, the signal was classified as an MPSO (+ or -). Otherwise, it was classified as symmetric (MPSOsym). We obtained similar classifications when the NEG-med and POS-med were calculated using 20% or 40% of the most positive and negative points. Moreover, the MPSO classification was always confirmed by a visual inspection.

### Mitral Cell Model

In **Figure 6B** and **Supporting Information S1**, we used a compartmental mitral cell model to infer the effects of inhibitory and excitatory oscillatory conductance injections into the tuft and lateral dendrites on the soma membrane potential. We used a mitral cell model that was developed by Migliore and Shepherd ([47]; ModelDB access number: 97263). Briefly, the model contained a soma, a primary dendrite with a tuft and two secondary dendrites. Spikes were generated by a sodium current, and a delayed rectifier potassium current induced the spike repolarization. A slow inactivating potassium A-current was also present.

Noisy oscillatory conductances were injected into the model as follows:
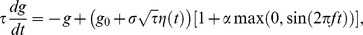
where *g* is the injected conductance, *τ* is the synaptic time constant, *g_0_* is the average conductance, *a* is the modulation amplitude, *f* is the modulation frequency, *η* is a white noise process and *σ* is the noise amplitude. The final injected current was calculated using the following equation:



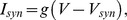
where *V_syn_* is the synaptic reversal potential. We simultaneously injected an excitatory conductance (*V_E_* = 0 mV, *τ_E_* = 3 ms) and an inhibitory conductance (*V_I_*_ = _ −80 mV, *τ_I_* = 10 ms) into the mitral cell tuft (see **Supporting Information S1** for other configurations of synaptic inputs). We used *g_I,0_* = 3 pS and *g_E,0_* = 1 pS, and *σ* was set at 50% of *g_E/I,0_*. Finally, we used *f = *2 Hz and *a* = 0.7.

Twelve seconds of real time were simulated. During the simulations, we monitored the membrane potential in the soma, the tuft and the middle of a secondary dendrite.

The simulations were performed on a standard Linux workstation using the NEURON simulator.

### Statistical Analysis

Statistical tests were performed using R combined with Python scripts. The level of significance was set at p<0.05 for all of the statistical tests (p<0.05*; p<0.01**; p<0.001***). The mean values are accompanied by SEM values. The McNemar test was applied to compare the proportion of oscillating cells between the control and odor stimulation conditions. The change in occurrence of an MPSO between the excitability states was compared using Fisher’s exact test. The proportion of cells of the various MPSO types (+, − or sym) was compared between the control and odor period and then between the excitability states using a χ^2^ test. The differences in the MPSO frequencies between the control and odor conditions were compared using a Mann-Whitney test. The correlations between the respiratory frequency and MPSO frequency were quantified using Pearson’s correlation coefficient. The M/T cell activity patterns were compared between two excitability levels, or between control and odor conditions, using a χ^2^ test, and the significant difference between the firing rates across the two excitability states was measured using a Mann-Whitney test.

## Supporting Information

Figure S1 **Inspection of membrane potential up and down levels for the different membrane potential slow oscillation (MPSO) types.** In order to facilitate the comparisons between cells, the membrane potential values of the up and down levels were computed relatively to a baseline (different for each cell and set to 0 in the figure), which depended on the MPSO type during the control period; the MPSO- baseline was the oscillation up level, the MPSO+ baseline was the oscillation down level and the NoMPSO baseline was the average membrane potential. For each cell, the same baseline was then subtracted from the MPSO up and down levels during the odor period, which facilitated the comparison of MPSOs between the control and odor periods. For the MPSO+ recordings, the up level was detected as the most positive membrane potential value. The down level value corresponded to the median of 30% of the most negative points. Conversely, the membrane potential up value of the MPSO- corresponded to the median of 30% of the most positive points, whereas the membrane potential down level corresponded to the membrane potential value of the negative peak. The mean membrane potential of the NoMPSO cells was determined by averaging the”cut-signal”. In **Figure S1**, the cells are sorted according to their MPSO types during the control and odor period. MPSO- is plotted in green, MPSO+ in red and NoMPSO in beige. Square, and diamond indicate down and up MPSO values respectively. *A. Cells with an MPSO- during the control and odor periods*. We observed a relatively stable level and amplitude of the oscillation when the odor was present, which suggested that the inhibition likely reached its maximum value in the control condition. *B and C. Cells without MPSOs during the control period with an MPSO- (B) or MPSO+ (C) during the odor period.* In B, the MPSO- is mainly a downward deviation from the baseline during the control period. This observation suggests that the emergence of an MPSO- may correspond to a rhythmic hyperpolarization of the membrane potential, most likely originating from synaptic inhibition. In contrast to C, the MPSO+ appears to be a combination of downward and upward deviations. The upward deviation may be supported by a rhythmic depolarization induced by the olfactory nerve excitatory input. The membrane potential down levels occurring at a more hyperpolarized potential than the control membrane potential may indicate that some intrinsic currents participate to shape the MPSO+, in particular, its down phase. *D. Cells with an MPSO+ before and during odor stimulation*. In this case, the odor strongly increases the up and down MPSO+ levels and the total amplitude. These modifications are consistent with increased excitatory input. *E. Cells with an MPSO+ during the control period and becoming MPSO- during the odor period.* The change in MPSO type is accompanied by a small but systematic decrease in both the up and down levels of the MPSO- relative to the MPSO+ during the control period. Both the shape and potential changes may be caused by a change in the excitatory and inhibitory input balance in favor of inhibition during odor presentation.
(TIF)

Supporting Information S1 **Model analysis demonstrating how a silent oscillation can induce a synchronized discharge.**
(DOC)

## References

[1] Lisman J (2005) The theta/gamma discrete phase code occuring during the hippocampal phase precession may be a more general brain coding scheme. Hippocampus 15: 913–922. 16161035 10.1002/hipo.20121

[2] Wang X (2010) Neurophysiological and computational principles of cortical rhythms in cognition. Physiol Rev 90: 1195–1268. 20664082 10.1152/physrev.00035.2008 PMC2923921

[3] Lisman J, Buzsáki G (2008) A neural coding scheme formed by the combined function of gamma and theta oscillations. Schizophr Bull 34: 974–980. 18559405 10.1093/schbul/sbn060 PMC2518638

[4] Steriade M (2006) Grouping of brain rhythms in corticothalamic systems. Neuroscience 137: 1087–1106. 16343791 10.1016/j.neuroscience.2005.10.029

[5] Adrian ED (1942) Olfactory reactions in the brain of the hedgehog. J Physiol 100: 459–473. 16991539 10.1113/jphysiol.1942.sp003955 PMC1393326

[6] Freeman WJ (1978) Spatial properties of an EEG event in the olfactory bulb and cortex. Electroencephalogr Clin Neurophysiol 44: 586–605. 77765 10.1016/0013-4694(78)90126-8

[7] Fontanini A, Spano P, Bower JM (2003) Ketamine-xylazine-induced slow (<1.5 Hz) oscillations in the rat piriform (olfactory) cortex are functionally correlated with respiration. J Neurosci 23: 7993–8001. 12954860 10.1523/JNEUROSCI.23-22-07993.2003 PMC6740491

[8] Buonviso N, Amat C, Litaudon P, Roux S, Royet JP, et al (2003) Rhythm sequence through the olfactory bulb layers during the time window of a respiratory cycle. Eur J Neurosci 17: 1811–1819. 12752780 10.1046/j.1460-9568.2003.02619.x

[9] Macrides F, Chorover SL (1972) Olfactory bulb units: activity correlated with inhalation cycles and odor quality. Science 175: 84–87. 5008584 10.1126/science.175.4017.84

[10] Onoda N, Mori K (1980) Depth distribution of temporal firing patterns in olfactory bulb related to air-intake cycles. J Neurophysiol 44: 29–39. 7420137 10.1152/jn.1980.44.1.29

[11] Ravel N, Caille D, Pager J (1987) A centrifugal respiratory modulation of olfactory bulb unit activity: a study on acute rat preparation. Exp Brain Res 65: 623–628. 3556489 10.1007/BF00235985

[12] Ravel N, Pager J (1990) Respiratory patterning of the rat olfactory bulb unit activity: nasal versus tracheal breathing. Neurosci Lett 115: 213–218. 2234500 10.1016/0304-3940(90)90457-k

[13] Chaput MA, Buonviso N, Berthommier F (1992) Temporal patterns in spontaneous and odour-evoked mitral cell discharges recorded in anaesthetized freely breathing animals. Eur J Neurosci 4: 813–822. 12106304 10.1111/j.1460-9568.1992.tb00191.x

[14] Aylwin MDLL, Díaz E, Maldonado PE (2005) Simultaneous single unit recording in the mitral cell layer of the rat olfactory bulb under nasal and tracheal breathing. Biol Res 38: 13–26. 15977406 10.4067/s0716-97602005000100003

[15] Charpak S, Mertz J, Beaurepaire E, Moreaux L, Delaney K (2001) Odor-evoked calcium signals in dendrites of rat mitral cells. Proc Natl Acad Sci U S A 98: 1230–1234. 11158622 10.1073/pnas.021422798 PMC14737

[16] Cang J, Isaacson JS (2003) In vivo whole-cell recording of odor-evoked synaptic transmission in the rat olfactory bulb. J Neurosci 23: 4108–4116. 12764098 10.1523/JNEUROSCI.23-10-04108.2003 PMC6741073

[17] Lehmkuhle MJ, Normann RA, Maynard EM (2006) Trial-by-trial discrimination of three enantiomer pairs by neural ensembles in mammalian olfactory bulb. J Neurophysiol 95: 1369–1379. 16306170 10.1152/jn.01334.2004

[18] Bathellier B, Buhl DL, Accolla R, Carleton A (2008) Dynamic ensemble odor coding in the mammalian olfactory bulb: sensory information at different timescales. Neuron 57: 586–598. 18304487 10.1016/j.neuron.2008.02.011

[19] Cury KM, Uchida N (2010) Robust odor coding via inhalation-coupled transient activity in the mammalian olfactory bulb. Neuron 68: 570–585. 21040855 10.1016/j.neuron.2010.09.040

[20] Kepecs A, Uchida N, Mainen ZF (2006) The sniff as a unit of olfactory processing. Chem Senses 31: 167–179. 16339265 10.1093/chemse/bjj016

[21] Shusterman R, Smear MC, Koulakov AA, Rinberg D (2011) Precise olfactory responses tile the sniff cycle. Nat Neurosci 14: 1039–1044. 21765422 10.1038/nn.2877 PMC13348895

[22] Schaefer AT, Angelo K, Spors H, Margrie TW (2006) Neuronal oscillations enhance stimulus discrimination by ensuring action potential precision. PLoS Biol 4: e163. 16689623 10.1371/journal.pbio.0040163 PMC1459879

[23] Litaudon P, Garcia S, Buonviso N (2008) Strong coupling between pyramidal cell activity and network oscillations in the olfactory cortex. Neuroscience 156: 781–787. 18790020 10.1016/j.neuroscience.2008.07.077

[24] Cenier T, David F, Litaudon P, Garcia S, Amat C, et al (2009) Respiration-gated formation of gamma and beta neural assemblies in the mammalian olfactory bulb. Eur J Neurosci 29: 921–930. 19291223 10.1111/j.1460-9568.2009.06651.x

[25] Margrie TW, Schaefer AT (2003) Theta oscillation coupled spike latencies yield computational vigour in a mammalian sensory system. J Physiol 546: 363–374. 12527724 10.1113/jphysiol.2002.031245 PMC2342519

[26] Phillips ME, Sachdev RN, Willhite DC, Shepherd GM (2012) Respiration drives network activity and modulates synaptic and circuit processing of lateral inhibition in the olfactory bulb. J Neurosci 32: 85–98. 22219272 10.1523/JNEUROSCI.4278-11.2012 PMC3566643

[27] Luo M, Katz LC (2001) Response correlation maps of neurons in the mammalian olfactory bulb. Neuron 32: 1165–1179. 11754845 10.1016/s0896-6273(01)00537-2

[28] Courtiol E, Amat C, Thévenet M, Messaoudi B, Garcia S, et al (2011) Reshaping of bulbar odor response by nasal flow rate in the rat. PLoS One 6: e16445. 21298064 10.1371/journal.pone.0016445 PMC3027679

[29] Grosmaitre X, Santarelli LC, Tan J, Luo M, Ma M (2007) Dual functions of mammalian olfactory sensory neurons as odor detectors and mechanical sensors. Nat Neurosci 10: 348–354. 17310245 10.1038/nn1856 PMC2227320

[30] Sobel EC, Tank DW (1993) Timing of odor stimulation does not alter patterning of olfactory bulb unit activity in freely breathing rats. J Neurophysiol 69: 1331–1337. 8492167 10.1152/jn.1993.69.4.1331

[31] Carey RM, Wachowiak M (2011) Effect of sniffing on the temporal structure of mitral/tufted cell output from the olfactory bulb. J Neurosci 31: 10615–10626. 21775605 10.1523/JNEUROSCI.1805-11.2011 PMC3159407

[32] Yuan Q, Knöpfel T (2006) Olfactory nerve stimulation-evoked mGluR1 slow potentials, oscillations, and calcium signaling in mouse olfactory bulb mitral cells. J Neurophysiol 95: 3097–3104. 16467433 10.1152/jn.00001.2006

[33] Balu R, Strowbridge BW (2007) Opposing inward and outward conductances regulate rebound discharges in olfactory mitral cells. J Neurophysiol 97: 1959–1968. 17151219 10.1152/jn.01115.2006

[34] Johnston J, Delaney KR (2010) Synaptic activation of T-type Ca2+ channels via mGluR activation in the primary dendrite of mitral cells. J Neurophysiol 103: 2557–2569. 20071628 10.1152/jn.00796.2009

[35] Balu R, Larimer P, Strowbridge BW (2004) Phasic stimuli evoke precisely timed spikes in intermittently discharging mitral cells. J Neurophysiol 92: 743–753. 15277594 10.1152/jn.00016.2004

[36] Carlson GC, Shipley MT, Keller A (2000) Long-lasting depolarizations in mitral cells of the rat olfactory bulb. J Neurosci 20: 2011–2021. 10684902 10.1523/JNEUROSCI.20-05-02011.2000 PMC6772924

[37] Schoppa NE, Westbrook GL (2001) Glomerulus-specific synchronization of mitral cells in the olfactory bulb. Neuron 31: 639–651. 11545722 10.1016/s0896-6273(01)00389-0

[38] Chaput MA (2000) EOG responses in anesthetized freely breathing rats. Chem Senses 25: 695–701. 11114147 10.1093/chemse/25.6.695

[39] Padmanabhan K, Urban NN (2010) Intrinsic biophysical diversity decorrelates neuronal firing while increasing information content. Nat Neurosci 13: 1276–1282. 20802489 10.1038/nn.2630 PMC2975253

[40] Debarbieux F, Audinat E, Charpak S (2003) Action potential propagation in dendrites of rat mitral cells in vivo. J Neurosci 23: 5553–5560. 12843256 10.1523/JNEUROSCI.23-13-05553.2003 PMC6741248

[41] Egger V (2008) Synaptic sodium spikes trigger long-lasting depolarizations and slow calcium entry in rat olfactory bulb granule cells. Eur J Neurosci 27: 2066–2075. 18412627 10.1111/j.1460-9568.2008.06170.x

[42] Yokoi M, Mori K, Nakanishi S (1995) Refinement of odor molecule tuning by dendrodendritic synaptic inhibition in the olfactory bulb. Proc Natl Acad Sci U S A 92: 3371–3375. 7724568 10.1073/pnas.92.8.3371 PMC42168

[43] Ennis M, Hamilton K, Hayar A (2007) Neurochemistry of the main olfactory system. In: Lajtha A, editor-in-chief. Handbook of neurochemistry and molecular neurobiology. Heidelberg: Springer. 137–204.

[44] Roux SG, Garcia S, Bertrand B, Cenier T, Vigouroux M, et al (2006) Respiratory cycle as time basis: an improved method for averaging olfactory neural events. J Neurosci Methods 152: 173–178. 16246424 10.1016/j.jneumeth.2005.09.004

[45] Garcia S, Fourcaud-Trocmé N (2009) Openelectrophy: an electrophysiological data- and analysis-sharing framework. Front Neuroinform 3: 14. 19521545 10.3389/neuro.11.014.2009 PMC2694696

[46] Wellis DP, Scott JW (1990) Intracellular responses of identified rat olfactory bulb interneurons to electrical and odor stimulation. J Neurophysiol 64: 932–947. 2230935 10.1152/jn.1990.64.3.932

[47] Migliore M, Shepherd GM (2008) Dendritic action potentials connect distributed dendrodendritic microcircuits. J Comput Neurosci 24: 207–221. 17674173 10.1007/s10827-007-0051-9 PMC3752904

[48] Tuckwell HC (1988) Introduction to Theoretical Neurobiology, Cambridge: Cambridge University Press.

