# Supplementary figures and images for: The Relationship between Respiration-Related Membrane Potential Slow Oscillations and Discharge Patterns in Mitral/Tufted Cells: What Are the Rules?
Source: PLoS One. 2012 Aug 31;7(8):e43964. doi: 10.1371/journal.pone.0043964 (PMC3432043; doi:10.1371/journal.pone.0043964)

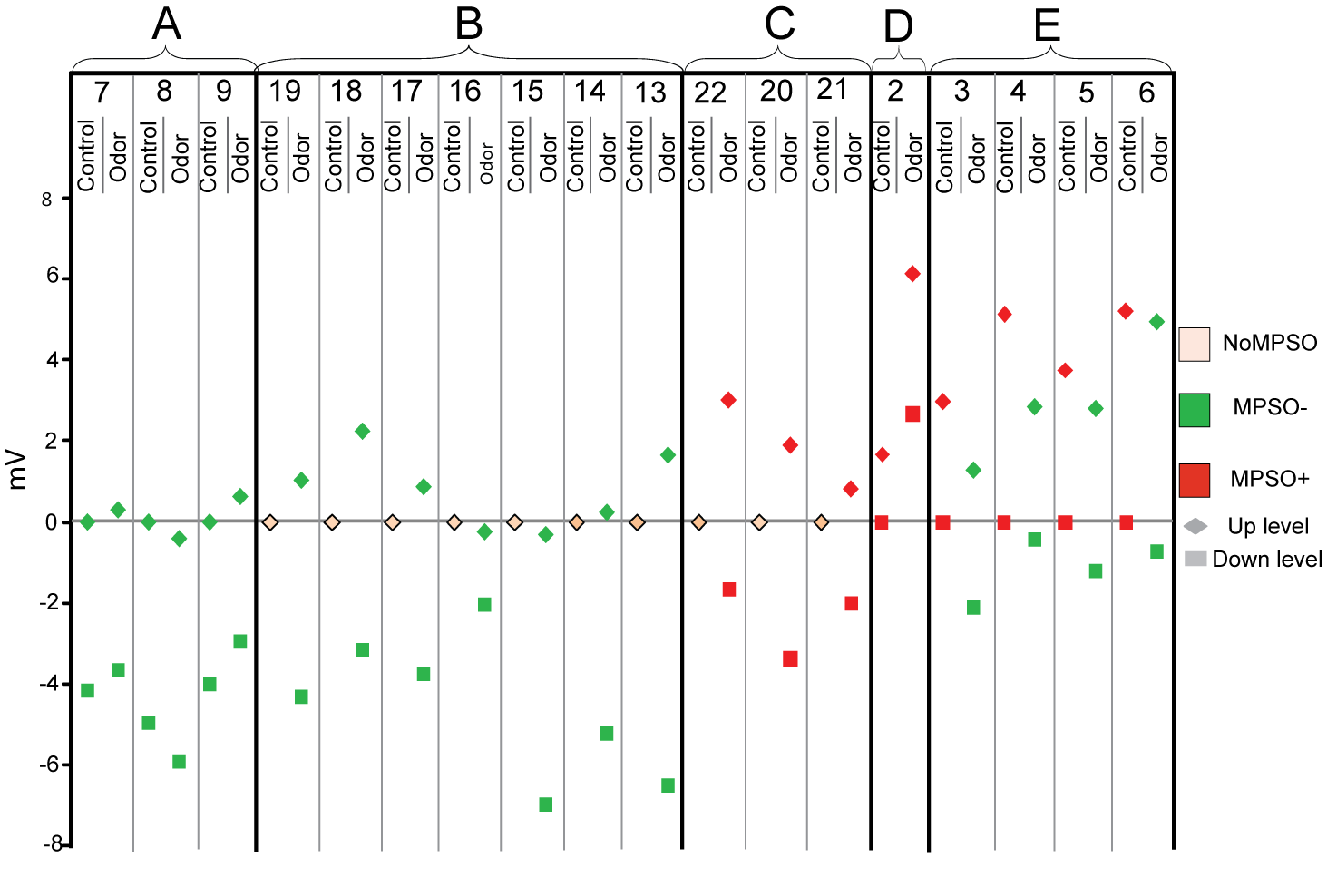

Supplement: Figure S1 — Inspection of membrane potential up and down levels for the different membrane potential slow oscillation (MPSO) types. In order to facilitate the comparisons between cells, the membrane potential values of the up and down levels were computed relatively to a baseline (different for each cell and set to 0 in the figure), which depended on the MPSO type during the control period; the MPSO- baseline was the oscillation up level, the MPSO+ baseline was the oscillation down level and the NoMPSO baseline was the average membrane potential. For each cell, the same baseline was then subtracted from the MPSO up and down levels during the odor period, which facilitated the comparison of MPSOs between the control and odor periods. For the MPSO+ recordings, the up level was detected as the most positive membrane potential value. The down level value corresponded to the median of 30% of the most negative points. Conversely, the membrane potential up value of the MPSO- corresponded to the median of 30% of the most positive points, whereas the membrane potential down level corresponded to the membrane potential value of the negative peak. The mean membrane potential of the NoMPSO cells was determined by averaging the”cut-signal”. In Figure S1, the cells are sorted according to their MPSO types during the control and odor period. MPSO- is plotted in green, MPSO+ in red and NoMPSO in beige. Square, and diamond indicate down and up MPSO values respectively. A. Cells with an MPSO- during the control and odor periods. We observed a relatively stable level and amplitude of the oscillation when the odor was present, which suggested that the inhibition likely reached its maximum value in the control condition. B and C. Cells without MPSOs during the control period with an MPSO- (B) or MPSO+ (C) during the odor period. In B, the MPSO- is mainly a downward deviation from the baseline during the control period. This observation suggests that the emergence of an MPSO- may correspond to a rhy [file pone.0043964.s001.tif]
