# Supplementary material for: The Relationship between Respiration-Related Membrane Potential Slow Oscillations and Discharge Patterns in Mitral/Tufted Cells: What Are the Rules?
Source: PLoS One. 2012 Aug 31;7(8):e43964. doi: 10.1371/journal.pone.0043964 (PMC3432043; doi:10.1371/journal.pone.0043964)
Supplement: Supporting Information S1 — Model analysis demonstrating how a silent oscillation can induce a synchronized discharge. (DOC) [file pone.0043964.s002.doc]

**Supporting Information S1**

**The relationship between respiration-related membrane potential slow oscillations and discharge patterns in mitral/tufted cells: What are the rules?**

Virginie Briffaud, Nicolas Fourcaud-Trocmé, Belkacem Messaouidi, Nathalie Buonviso and Corine Amat

**Institution :** Lyon Neuroscience Research Center (CRNL), Team Olfaction: from coding to memory, CNRS UMR 5292 - INSERM U1028 - Université Lyon 1, Lyon, France

Here, we present a model analysis demonstrating how a silent oscillation can induce a synchronized discharge.

Our first question was: how can a synaptic oscillation be masked then revealed by hyperpolarization? If we consider that a neuron receives two synaptic conductances with an identical time-dependence, we can write the total synaptic current as:

*I_syn_(t) = g_E_ f (t)(V - E_E_) + g_I_ f(t)(V – E_I_)*,

where *g_E_* , *E_E_*, resp. *g_I_*, *E_I_*, are the excitatory, resp. inhibitory, conductance amplitude and reversal potential respectively. *f(t)* describes the conductance time-dependence, which is identical for both excitation and inhibition. This equation can be rewritten as:

*I_syn_(t) = g_mixed_ f(t)(V – E_mixed_)*,

where *g_mixed_ = g_E_ + g_I_* and $E_{mixed}=\frac{g_{E}E_{E}+g_{I}E_{I}}{g_{I}+g_{E}}$.

This equation clearly demonstrates that if the neuron membrane potential *V* is maintained equal to *E_mixed_*, the total synaptic current is null; however, there is an underlying fluctuation of synaptic conductances. Then, if *f(t)* is an oscillation, a silent oscillation can be revealed by membrane excitability changes, such as hyperpolarization or depolarization.

The next question is: how can such a silent oscillation cause a synchronized discharge? Neuronal synaptic inputs are the sum of many small synaptic inputs. Using the diffusion approximation (for example, see [48]), this sum can be described by a mean synaptic conductance plus a noise, which has an amplitude that increases with the conductance mean. In this case, during a silent oscillation, the amplitude of random fluctuations of *I_syn_*, which are caused by the random arrival of synaptic inputs, is maximal when *f(t)* is maximal. This process can induce neuronal rhythmic discharge. Indeed, assuming that the membrane potential is below the action potential threshold, larger *_Isyn_* fluctuations enable the action potential threshold to be reached more easily and frequently, inducing a transient increase of the neuronal firing rate while its average membrane potential is still flat.

We confirmed this hypothesis using an integrate-and-fire model with oscillating synaptic inputs as follows:

$$\begin{matrix} C\frac{dV}{dt}=g_{L}\left(V-E_{L}\right)+g_{E}\left(V-E_{E}\right)+g_{I}\left(V-E_{I}\right) \\ \tau_{E}\frac{dg_{E}}{dt}=-g_{E}+\left(g_{E,0}+\sigma_{E}\sqrt{\tau_{E}}\eta_{E}\left(t\right)\right)\left(1+2a_{E}\left(\max\left(0,\cos\left(2\pi vt+\varphi_{E}\right)\right)-0.5\right)\right) \\ \tau_{I}\frac{dg_{I}}{dt}=-g_{I}+\left(g_{I,0}+\sigma_{I}\sqrt{\tau_{I}}\eta_{I}\left(t\right)\right)\left(1+2a_{I}\left(\max\left(0,\cos\left(2\pi vt+\varphi_{I}\right)\right)-0.5\right)\right), \end{matrix}$$

where *C = 1* ηF is the membrane capacitance; *g_L_ =* 0.1 µS and *E_L_ = E_mixed_* = -53.3 mV are the leak conductance and reversal potential, respectively; *E_E_* = 0 mV; and *E_I_* = -80 mV. The noisy synaptic inputs were filtered using the synaptic time constants *τ_E_* = 3 ms and *τ_I_* = 10 ms. The average synaptic inputs were *g_E,0_* = 1 µS and *g_I, 0_* = 2 µS; *η*_E_*(t)* and *η_I_(t)* were independent Gaussian noises. The noise amplitudes were *σ_E_* = 0.2 µS and *σ_I_* = 0.4 µS. We determined the modulation amplitudes as a fraction of the average conductances, *α_E_* = *α_I_* = 0.7, and the phase shifts *Φ_E_* and *Φ_I_* were adjusted to compensate for the phase lags caused by the filtering. Finally, the spike threshold was set to -50 mV, and the membrane potential was reset to *-*65 mV after a 5 ms absolute refractory period. Of note, in the model, we set the I/E transition at the middle of the respiratory cycle.

We plotted two traces obtained by the simulation of this model in **Figure S1-1A**. On the right, without hyperpolarization, we observed that the model membrane potential presented no intracellular slow oscillation (as assessed by the Fourier spectrum, *lower left panel*), whereas the spiking activity was rhythmic (*lower right panel*). The absence of intracellular oscillation is observed because, with the exception of the additional noise term, we are in the case where the membrane potential is equal to the oscillation reversal potential as described previously. However, because the amplitude of the additional noise is oscillating, the model is more prone to spike at a specific respiratory cycle phase. It is noteworthy that in the hyperpolarized case (*left panels*), the conductance oscillation was apparent because the membrane potential no longer equaled *E_mixed_*.

In **Figure S1-1B**, we confirmed that the absence of intracellular oscillations was not a side effect of the neuron spiking dynamics by simply removing the spiking mechanism of the model i.e., by not considering the spike threshold and by considering the membrane potential as always being the subthreshold. In this case, there was still no intracellular oscillation. We observed that the membrane potential standard deviation along an average input cycle oscillated as expected (data not shown), and, in the presence of the spiking mechanism, this oscillation led to rhythmic spiking.


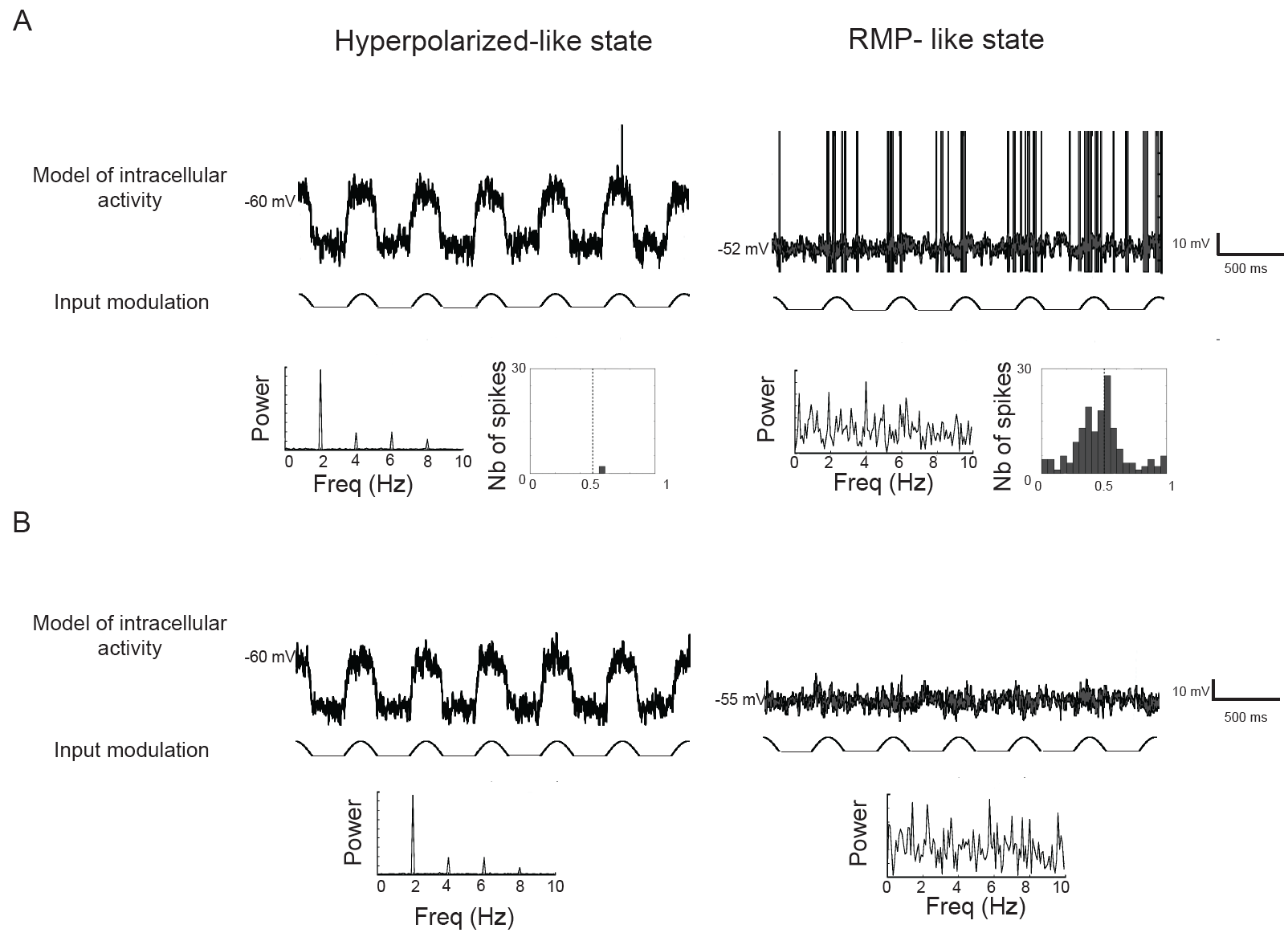
**Figure S1-1: An integrate-and-fire model demonstrating discharge synchronization was induced by a silent oscillation**

1. *A model response to an oscillatory mixture of excitatory and inhibitory synaptic inputs with (left panels) or without (right panels) an additional hyperpolarizing current*. Top: model intracellular activity; middle: simulated input; bottom: the Fourier power spectrum plot of the membrane potential and the resulting respiratory cycle-triggered spike histogram with the inspiration/expiration transition (at 0.5) marked by a vertical dotted line. RMP: resting membrane potential, Freq: frequency
2. *Same as A, following the removal of the model spiking threshold.* The simulation parameter details are provided in the text. Top: intracellular activity simulation; middle: simulated input, bottom: the Fourier power spectrum plot of the membrane potential.

Furthermore, we wanted to confirm whether this mechanism was still valid in a detailed, multi-compartment mitral cell model. We injected the same synaptic currents into a mitral cell model as previously described by Migliore and Shepherd ([47], ModelDB access number: 97263).

First, we injected the synaptic currents only in the mitral tuft. This injection can be seen as a mixture of excitation from the olfactory receptor neurons and inhibition from the periglomerular cells. Because *E_L_* = -60 mV in the cell, we used *g_I,0_ =* 3 *g_E,0_=* 3 pS to maintain the condition *E_mixed_ = E_L_*, and the noise was set to 50% of the average synaptic conductances. In **Figure S1-2**, we plotted the traces that were recorded in the mitral model tuft (the soma traces are plotted in **Figure 6B**). Although we did not observe any intracellular oscillations, we observed rhythmic spiking. All of the observations made in the single compartment model were thus still valid in this case. Simply, the tuft high noise level was strongly attenuated in the soma because of its larger size and the passive filtering of the membrane potential fluctuations along the main apical dendrite.

Second, because mitral cells do not generally receive inhibitory inputs only in their tuft, we split the inhibitory input between the tuft and lateral dendrites. In the lateral dendrites, the inhibitory input was injected into 5 evenly spaced locations along each dendrite. Because the attenuation of the synaptic inputs was different from each synapse to the soma, we manually adjusted the level of lateral dendrite inhibition to compensate for the unbalanced tuft inhibition. **Figure S1-3** demonstrates an example of such a simulation with, in the tuft, *g_I,0_ = g_E,0_ =* 0.8 pS, and in the lateral dendrites (5 locations in each dendrite), *g_I,0_ =* 0.2 pS. The noise was set to 20% of the average synaptic conductances. Moreover, the inhibitory input in the lateral dendrites was shifted by *Φ*= 0.4. Based on these parameters, we again failed to observe an MPSO at the soma (**Figure S1-3B**, *right panels*), while an MPSO was clearly visible in the tuft (**Figure S1-3A,** *right panels*). Finally, it is noteworthy that when the lateral and tuft inhibitions were mixed, it was very difficult to cause the cells to be well-synchronized due to their different propagation dynamics to the soma; this mixture of inhibitions frequently resulted in rhythmic Sc patterns rather than rhythmic S+ patterns, which was shown in previous models.


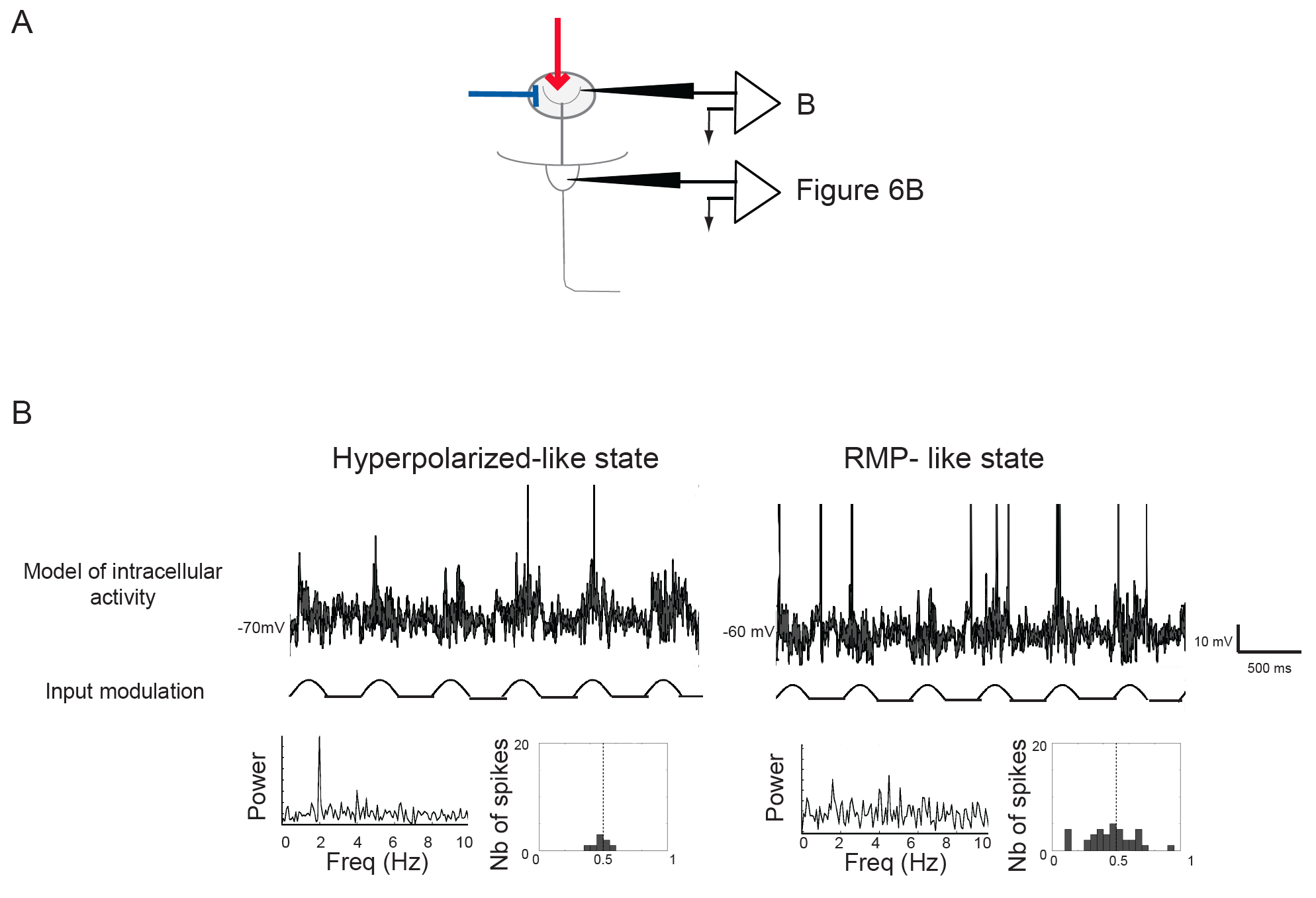


**Figure S1-2: Simulation of a multi-compartment mitral cell model with synaptic inputs only in the tuft. The intracellular activity is recorded in the tuft.**

1. *A schematic representation of the input and recording locations.* The excitatory input location is indicated with a red arrow, whereas the inhibitory input is represented by the blue line.
2. *The intracellular simulated activities are obtained at the two following excitability levels: the resting membrane potential (RMP)-like state (right panels) and a hyperpolarized-like state (left panels) when a negative current was injected into the soma*. Top: tuft intracellular activity simulations; middle: simulated input; bottom: Fourier power spectrum plot of membrane potential, and the respiratory cycle-triggered spike histogram with the inspiration/expiration transition (at 0.5) indicated by a vertical dotted line. The simulation parameter details are provided in the text.

**
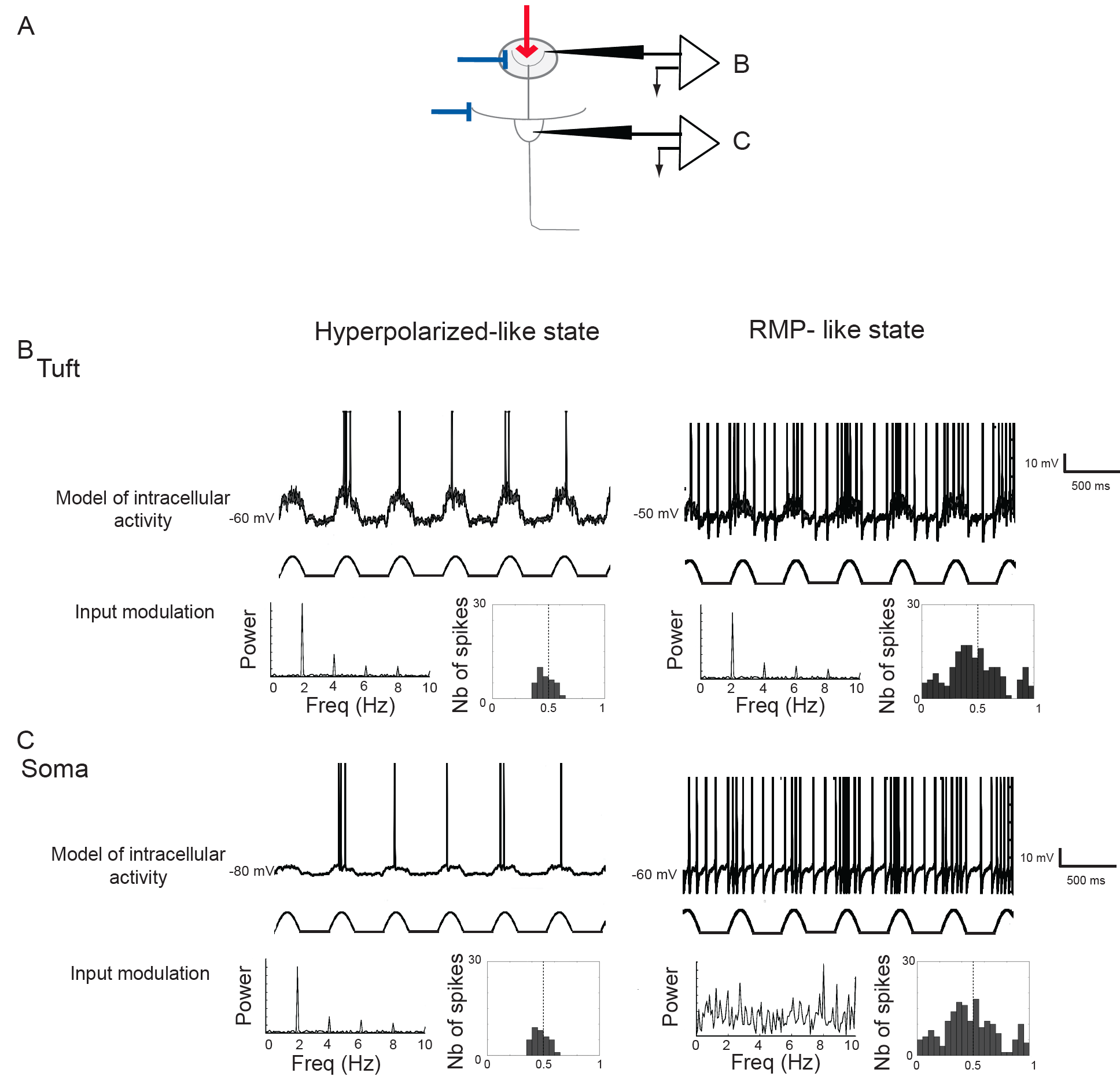
**

**Figure S1-3:** **Simulation of a multi-compartment mitral cell model with excitatory synaptic input in the tuft and inhibitory input in both the tuft and lateral dendrites.**

1. *A schematic representation of the input and recording locations.* The excitatory input location is indicated by the red arrow, whereas the inhibitory inputs are represented by the blue lines.
2. *Simulation of the mitral tuft activity at the resting membrane potential (RMP)-like state (right panels) and hyperpolarized-like state (left panels)*. Top: intracellular activity simulation; middle: simulated input; bottom: Fourier power spectrum plot of membrane potential and the resulting respiratory cycle-triggered spike histogram with the inspiration/expiration transition (at 0.5) indicated by a vertical dotted line.
3. *The same simulation as in B, but the intracellular activity is recorded in the soma*. The simulation parameter details are provided in the text. These simulations were obtained simultaneously with those in B.

**References**:

[47] Migliore M, Shepherd GM (2008) Dendritic action potentials connect distributed

dendrodendritic microcircuits. J Comput Neurosci 24: 207-221.

[48] Tuckwell HC (1988) Introduction to Theoretical Neurobiology, Cambridge: Cambridge University Press.
